# Autophagy in emerging and highly concerned severe zoonotic infectious diseases

**DOI:** 10.3389/fimmu.2026.1761571

**Published:** 2026-01-27

**Authors:** Yubo Qi, Lingjie Wang, Shengping Wu, Chi Meng, Yuefeng Chu, Yulong Yin, Hanwei Jiao

**Affiliations:** 1 The College of Veterinary Medicine, Southwest University, Chongqing, China; 2 State Key Laboratory for Animal Disease Control and Prevention, College of Veterinary Medicine, Lanzhou University, Lanzhou Veterinary Research Institute, Chinese Academy of Agricultural Sciences, Lanzhou, China; 3 Institute of Subtropical Agriculture, Chinese Academy of Sciences, Changsha, China

**Keywords:** autophagy, bacterial infection, regulatory mechanisms, viral infection, zoonotic infectious diseases

## Abstract

Autophagy is a conserved cellular process that mediates degradation of damaged organelles, misfolded proteins, and invading pathogens, playing critical roles in intracellular homeostasis and immune regulation. Given that over 70% of infectious diseases and 60% of emerging infectious diseases are zoonotic, posing a major threat to global health, this review aims to summarize the cellular and molecular mechanisms underlying the crosstalk between autophagy and key zoonotic pathogens. We comprehensively retrieved relevant research literature from the PubMed, Web of Science, and Scopus databases (with the retrieval deadline set as December 2025), using core keywords including autophagy, zoonoses, and pathogen-host interactions. The inclusion criteria were original studies and high-quality reviews focusing on molecular mechanisms or clinical translational potential. Finally, a total of 216 core literatures were included for comprehensive analysis. This review is a narrative overview with comprehensive coverage, aiming to systematically summarize the research progress of autophagy in zoonoses, rather than a systematic meta-analysis strictly adhering to the PRISMA guidelines. Key findings include (1): Autophagy can restrict the replication of zoonotic pathogens such as influenza virus and Brucella by mediating their degradation; (2) Some pathogens have evolved strategies to hijack or inhibit autophagy for survival; (3) Several autophagy-related molecules (e.g., ATG5, Beclin-1) have been identified as potential targets for zoonoses prevention and treatment. This review highlights the dual role of autophagy in zoonotic infections and its potential as a therapeutic target. However, further studies are needed to clarify species-specific differences in autophagy regulation and develop targeted interventions. These insights may provide new avenues for the prevention and treatment of severe zoonotic diseases.

## 1 Introduction

In recent years, an increasingly prominent threat to global public health security and social development has been posed by emerging and highly concerned severe zoonotic infectious diseases. Not only do Severe Acute Respiratory Syndrome (SARS), Coronavirus Disease 2019 (COVID-19), Middle East Respiratory Syndrome (MERS), Ebola hemorrhagic fever, Brucellosis, and other such diseases lead to massive human infections and deaths, but they also trigger severe economic losses and widespread public panic (1–3).

More than 70% of infectious diseases, as reported in relevant studies, are classified as zoonotic, and 60% of emerging infectious diseases fall into this category as well. It is this set of compelling data that clearly highlights zoonotic infectious diseases as a core focus in global efforts to prevent and control infectious diseases—an urgent need for in-depth research to address the challenges they entail is thus evident (4–7).

Against this critical backdrop, a top priority for researchers worldwide has become exploring the cellular and molecular mechanisms underlying the occurrence, development, and pathogenic processes of these diseases. Among the numerous intricate cellular processes, it is autophagy—a highly conserved “self-eating” phenomenon inherent in eukaryotic cells—that has gradually emerged as a key research direction in the study of infectious diseases. Via the fusion of autophagosomes and lysosomes, autophagy mediates the degradation and recycling of cytoplasmic components, intracellular organelles, and proteins; moreover, it plays an indispensable role in maintaining intracellular homeostasis. Of greater significance is the complex regulatory effect exerted by autophagy in the intricate interaction between pathogenic microorganisms and host cells—an effect that may either inhibit or promote the survival and replication of pathogens.

A solid theoretical and experimental foundation for understanding autophagy’s role in infectious diseases has been laid by previous studies, with macroautophagy being the primary focus in this context. Tightly regulated by autophagy-related genes (ATG) and multiple signaling pathways (e.g., PI3K, mTOR), the autophagic process can be induced by factors such as microbial infection, organelle damage, and protein misfolding. However, fully elucidated remain neither the specific role of autophagy in different emerging severe zoonotic infectious diseases nor the intricate regulatory networks involved. Notably, substantial variations exist in the interaction patterns between autophagy and different pathogens: while host autophagy counteracts SARS-CoV-2 infection by degrading viral particles, the virus has evolved strategies to manipulate the autophagic pathway for its own replication; Brucella induces a non-classical autophagic pathway distinct from the classical process to achieve intracellular survival; and Anthrax toxin triggers autophagy, which may be involved both in the pathogenesis of Anthrax and the host’s defense against bacterial infection.

To date, certain valuable findings have been yielded by research on the role of autophagy in individual types of zoonotic infectious diseases. However, a comprehensive, systematic summary and comparative analysis of autophagy’s function and mechanism across these diverse diseases is lacking. This gap not only hinders the formation of a unified and in-depth understanding of the overall relationship between autophagy and zoonotic infectious diseases but also limits the development of novel prevention and treatment strategies targeting the autophagic pathway. Therefore, of great theoretical significance and practical value is the task of systematically sorting out the research progress of autophagy in these diseases, clarifying its specific regulatory mechanisms, and exploring its potential as a therapeutic target.

In this review, we aim to address three key questions: First, what are the specific regulatory mechanisms of autophagy in major emerging and highly concerned severe zoonotic infectious diseases, including COVID-19, SARS, MERS, Ebola hemorrhagic fever, Dengue fever, AIDS, Rotavirus disease, Zika virus infection, Brucellosis, Anthrax, Avian influenza, and Rabies? Second, what are the similarities and differences in the role of autophagy between viral and bacterial zoonotic infectious diseases, and what are the underlying reasons for these differences? Third, what is the potential of targeting autophagy for the prevention and treatment of these zoonotic infectious diseases, and what are the current research progress, existing challenges, and future development directions in this field? By answering these questions, we expect this review to provide a comprehensive and up-to-date reference for researchers engaged in the study of autophagy and zoonotic infectious diseases and further contribute to the development of more effective prevention and treatment measures to combat these devastating diseases.

## 2 Typical characteristics of molecular regulation of autophagy

**Figure 1:**
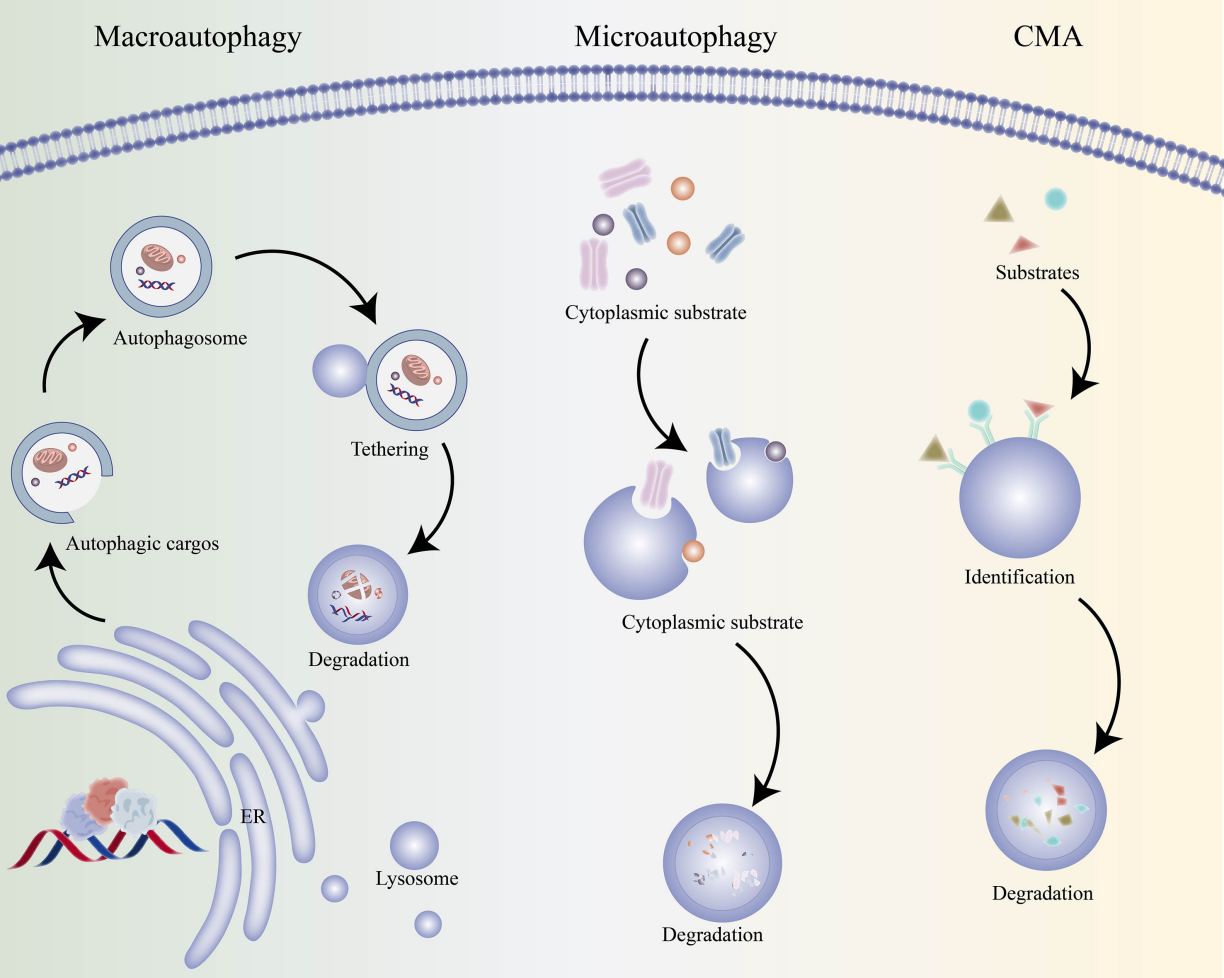
Three types of autophagy pathways. Macroautophagy. The most common autophagy type. It starts with induction, nucleation, and elongation, where the endoplasmic reticulum (ER) helps form the initial structure of the phagophore. Through substrate recognition, the phagophore engulfs targets (e.g., mitochondria). After closure, it matures into an autophagosome. The autophagosome then undergoes tethering and fusion with a lysosome, delivering contents for degradation. By - products are recycled for cellular use. Microautophagy. A more direct process. After substrate recognition, the lysosomal membrane undergoes invagination to engulf cytoplasmic substrates. Next, fission releases the engulfed portion into the lysosomal lumen for degradation. It mainly breaks down small molecules or organelle fragments. Chaperone - Mediated Autophagy (CMA). Relies on molecular chaperones. First, recognition occurs: chaperones bind substrate proteins with specific amino - acid sequences. Then, via translocation, substrates are moved to the lysosomal membrane and into the lumen for degradation. CMA primarily degrades soluble cytoplasmic proteins, playing a key role in cellular stress responses and protein homeostasis.

**Figure 2:**
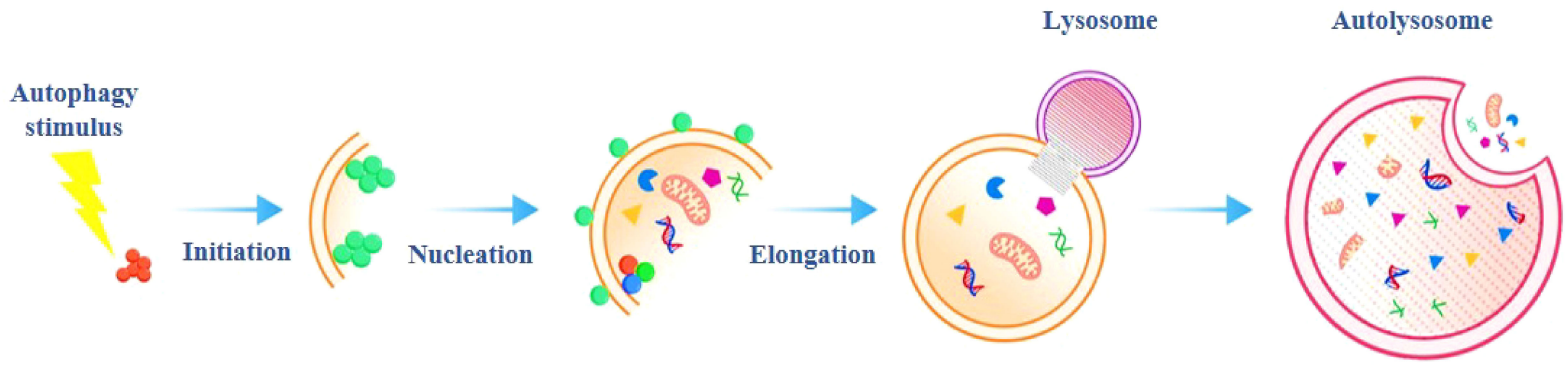
The classical autophagy pathway: From initiation to degradation. This infographic outlines the sequential steps of the autophagy pathway, a vital cellular process for recycling and maintaining homeostasis: 1).Initiation: The process begins with the formation of an isolation membrane (or phagophore), which starts to engulf cytoplasmic components (e.g., damaged organelles, misfolded proteins). 2).Vesicle Elongation: The isolation membrane expands and elongates, surrounding more targets (e.g., mitochondria, ribosomes). This step shapes the membrane into a crescent - like structure. 3).Maturation: The elongated membrane closes to form a double - membraned autophagosome, fully sequestering the cytoplasmic cargo for degradation. 4).Docking and Fusion: The autophagosome “docks” and fuses with a lysosome. Lysosomes supply acidic hydrolases that break down the autophagosome’s contents. 5).Vesicle Breakdown and Degradation: Inside the fused structure (autolysosome), the sequestered materials are degraded. The resulting nutrients (amino acids, sugars, etc.) are released back into the cytoplasm for reuse, supporting cellular metabolism or repair. In short, autophagy enables cells to remove dysfunctional components and recycle resources—key for survival during stress, development, and maintaining overall cellular health.

Autophagy refers to a double-layer membrane that falls off from the ribosome free attachment area of the rough endoplasmic reticulum, wraps some cytoplasmic and intracellular organelles, proteins and other components that need to be degraded to form autophagosomes, and fuses with lysosomes to form autophagosomes, which degrade the contents, so as to realize the metabolic needs of cells and the renewal of some organelles (3, 8–10). Autophagy includes basic autophagy under physiological conditions and induced autophagy under stress conditions, the former is the self-protection mechanism of cells, which is beneficial to the growth and development of cells, protects cells from metabolic stress and oxidative damage, and plays an important role in maintaining intracellular homeostasis and the synthesis, degradation and recycling of cell products. However, excessive autophagy may lead to metabolic stress, degradation of cell components, and even cell death. Studies have shown that autophagy can play an important role in a variety of physiological and pathological processes (11, 12). Autophagy can be divided into the following three types according to the different ways in which cellular substances are transported to lysosomes, they are macroautophagy, microautophagy, and chaperone mediated autophagy (CMA) (**Figure 1**). Generally speaking, autophagy refers to macroautophagy, microautophagy directly engulfs specific organelles through the deformation of lysosome or vacuole surface, CMA is some molecular chaperones, such as HSP70, which can help the translocation of unfolded proteins into lysosomes (13, 14).The essence of autophagy is actually membrane rearrangement in cells, its occurrence process can be divided into the following four stages: initiation of autophagy, vesicle elongation, formation of isolation membrane and autophagosome, autophagosome fusion with lysosomes to form autolysosome (**Figure 2**). After receiving the autophagy induction signal, the cell forms a small “liposome” like membrane structure somewhere in the cytoplasm, and then expands continuously, but it is not spherical, but flat, like a bowl composed of two lipid bilayers, which can be observed under the electron microscope, it is called phagophore, which is the first biomarkers of autophagy (15). Phagophore continues to elongate, taking all the components in the cytoplasm, including organelles, into the bowl, and then closing into a closed spherical autophagosome, which is the second hard evidence of autophagy. Autophagosome has two typical characteristics: one is the bilayer membrane, the other is the inclusion of cytoplasmic components, such as mitochondria, endoplasmic reticulum fragments and so on (16). After the autophagosome is formed, it can fuse with the phagocytic vesicles, swallowing vesicles and endosomes of the cells (this is not inevitable). During the formation of autolysosome, the inner membrane of autophagosome is degraded by lysosomal enzymes, the contents of the two are integrated, the cargo in autophagosome are also degraded, and the products (amino acids, fatty acids, etc.) are transported to the cytoplasm for cell reuse, while the residue is either discharged out of the cell or retained in the cytoplasm (15, 17, 18). Once the process of autophagy is initiated, it must be stopped in time after the crisis, otherwise, its non-specific capture of cytoplasmic components will lead to irreversible damage to cells, this also reminds us that we must observe autophagy dynamically, the results of any cross-sectional study are not enough to evaluate the activity of autophagy. At present, it has been reported that many factors can induce autophagy, such as starvation, growth factor deficiency, microbial infection, organelle damage, protein folding error or aggregation, DNA damage, radiotherapy, chemotherapy and so on (19). In the process of autophagy, a variety of autophagy related genes (ATG) can regulate and control the different stages of autophagy formation. So far, more than 40 genes encoding ATG protein have been identified in yeast, and most of them are highly conserved between yeast and mammals (20, 21). In mammalian cells, autophagy is regulated by about 20 core ATG genes, which are continuously recruited near vacuoles and assembled to form pre-autophagosomal structure (PAS) (**Table 1**).

**Table 1:** Core ATG genes involved in mammalian autophagy regulation.

Category	Specific genes/Families	Main functions	Conservation (vs. Yeast)
Initiation & Signaling	ATG1/ULK1	Forms the ULK1 complex, integrates autophagy initiation signals (e.g., interacts with mTOR kinase)	
	ATG13	Core component of the ULK1 complex, regulates ULK1 kinase activity	Conserved with yeast Atg13
	ATG17/FIP200	FIP200 in mammals replaces yeast Atg17, involved in ULK1 complex assembly and localization	Functionally conserved, partially conserved in sequence
Nucleation	ATG6/Beclin1	Forms complexes with Class III PI3K, involved in pre-autophagosomal structure (PAS) nucleation	Highly conserved with yeast Atg6
	ATG14	Binds to the Beclin1 complex, promotes its localization to PAS, regulates autophagy-specific nucleation	Conserved with yeast Atg14
Ubiquitin-like System	ATG5	Conjugates with ATG12 to form the ATG5-ATG12 complex, involved in autophagosomal membrane elongation	Highly conserved with yeast Atg5
	ATG7	E1-like enzyme, catalyzes ubiquitin-like conjugation reactions of ATG8 and ATG12	Highly conserved with yeast Atg7
	ATG10	E2-like enzyme, participates in the conjugation of ATG12 to ATG5	Conserved with yeast Atg10
	ATG12	Covalently binds to ATG5 to form a complex, providing structural support for autophagosomal membrane elongation	Highly conserved with yeast Atg12
ATG8 Family	ATG8/LC3 (LC3A, LC3B, LC3C)	Processed to bind autophagosomal membranes, involved in membrane elongation and cargo recognition	Conserved with yeast Atg8 family
	GABARAP Family (GABARAP, GABARAPL1)	Similar to LC3 in function, involved in autophagosome maturation and lysosome fusion	Conserved with yeast Atg8 family
Other Core Genes	ATG9	The only transmembrane ATG protein, involved in autophagosomal membrane source and transport	Highly conserved with yeast Atg9
	ATG2	Interacts with ATG9, regulates membrane structure elongation and fusion	Conserved with yeast Atg2
	ATG18/WIPI (WIPI1, WIPI2)	Binds to phosphatidylinositol, involved in PAS formation and autophagosomal membrane assembly	Conserved with yeast Atg18
	ATG4	Cysteine protease, processes LC3 precursors to maturity (e.g., cleaves pro-LC3 to generate LC3-I)	Highly conserved with yeast Atg4
	ATG16L1	Binds to the ATG5-ATG12 complex, forms a larger complex, regulates LC3 localization on autophagosomal membranes	Conserved with yeast Atg16

The regulation of autophagy is complex, the pathways that transmit autophagy signals are relatively certain: class I PI3K pathway binds to insulin receptor substrate (IRS) and receives signals from insulin receptors (22). MTOR pathway (mammalian target of rapamycin) the homologous gene of mTOR in human is FK506 binding protein 12 rapamycin associated protein 1 (Frap1), which is a serine/threonine protein kinase, it can receive a variety of upstream signals, such as class I PI3K, IGF-1/2 and MAPK, and can feel the changes of nutrition and energy, for this pathway, rapamycin is the most typical and commonly used autophagy agonist (23). Class III PI3K belongs to the activated signal pathway and can be blocked by 3-Methyladenine (3-MA) (22, 24, 25). We summarized the commonly used autophagy inducers and inhibitors, as shown in **Table 2**.

**Table 2:** Autophagy pathway - regulating drugs.

Autophagy stage	Activator	Target/Pathway of action	Inhibitor	Target/Pathway of action
Initiation	A - 769662、Calorie restriction、H_2_S、Resveratrol、Physical exercise、Simvastatin	AMPK	Cardiac glycosides	Na^+^/K^+^ - ATPase
	Caloric restriction、Everolimus、Physical exercise、Torins	mTORC1	Compound C	AMPK
	Antibacterial agents、Carbon monoxide、Melatonin	ROS	Edavarone、Mdivi - 1	ROS
	IFNγ	MAPK	3 - MA、LY294002、Wortmannin、VPS34 - IN1	VPS34
	Carbamazepine、Lithium	Ins(1,4,5)P_3_	NSC185084	ATG4B
	BECN1 - activating peptide	BECN1		
	CRM(Hydroxycitrate、Resveratrol、Spermidine)	BECN1		
	RBDO5831、SCH58261、Retinoic acid	Unknown		
	Fostriecin、 Trichostatin A、Vorinostat	Unknown		
Nucleation		No separate corresponding	3 - MA、LY294002、Wortmannin、VPS34 - IN1	VPS34
Elongation		No separate corresponding	MRB0703、MRT67307、SBI - 0206965	ULK1
Fusion		No separate corresponding		No separate corresponding
Degradation		No separate corresponding	Baf A1、Chloroquine、Ly05	Lysosomal inhibition

Under physiological conditions, autophagy activity is usually low, but it will be significantly up-regulated under stimulating factors. In addition, autophagy inhibition is also related to some diseases, choosing an ideal detection method is very important for autophagy research (26). Common methods for observing and detecting autophagy include: transmission electron microscope (TEM) directly observe the morphological changes of autophagy at different stages, phagophore is characterized by crescent or cup-shaped, double-layer or multilayer films, with a tendency to surround cytoplasmic components, autophagosome is characterized by double-layer or multilayer vacuolar structures containing cytoplasmic components, such as mitochondria, endoplasmic reticulum, ribosomes and so on, autolysosome is characterized by monolayer membrane and degradation of cytoplasmic components (27). GFP-LC3 single fluorescence or mRFP-GFP-LC3 double fluorescence was observed by fluorescence microscope to analyze autophagic flux, when autophagosomes are formed, green or yellow fluorescent spots are formed under the fluorescence microscope, when autophagosome and lysosome begin to fuse, GFP fluorescence decreases, after autolysoosome is formed, acidic environment quenches GFP fluorescence and presents red fluorescence (28). The level of autophagy can be estimated by detecting the ratio of LC3-II/I by western blot, when autophagy occurs, cytoplasmic LC3-I will enzymolysis a small segment of polypeptide, and then combine with PE to transform into membrane LC3-II (29). Using western blot to detect the expression of p62 protein can also evaluate the level of autophagy, in the process of autophagosome formation, p62, as a bridge between LC3 and polyubiquitinated protein, is selectively encapsulated into autophagosome, and then degraded by protein hydrolase in autolysosome (30). Therefore, the expression of p62 protein is negatively correlated with autophagy activity.

## 3 Physiological functions of autophagy

Autophagy is a highly conserved cellular degradation process in eukaryotes. It maintains cellular homeostasis by encapsulating and eliminating intracellular substances (such as damaged organelles and protein aggregates) and plays a central role in various physiological processes. Its physiological functions are elaborated from six aspects as follows.

### 3.1 Maintaining cellular homeostasis

#### 3.1.1 Participating in energy regulation

When nutrients are scarce or energy demand surges, autophagy is activated. It generates metabolic substrates such as amino acids and fatty acids by degrading macromolecules like proteins and lipids, providing energy for cells and maintaining energy balance. For example, in a state of hunger, liver cells decompose glycogen and lipid droplets through autophagy to meet basic metabolic needs (31). This process is crucial for maintaining cell survival under stress conditions and is one of the core regulatory mechanisms of cellular energy homeostasis.

#### 3.1.2 Participating in the turnover and reutilization of intracellular substances

Autophagy continuously removes aged or damaged biomolecules and organelles (such as mitochondria and endoplasmic reticulum), degrades them into small molecules, which are reused for biosynthesis, realizing material circulation. For instance, during the maturation of red blood cells, autophagy removes excess mitochondria; liver cells degrade abnormal proteins through autophagy to maintain proteome homeostasis (32). This turnover mechanism ensures the renewal of cellular components and is the basis for maintaining cellular functions.

### 3.2 Adapting to metabolic stress

Autophagy is a key mechanism for cells to cope with metabolic stress (such as hypoxia, oxidative stress, and nutrient deprivation). Under hypoxic conditions, autophagy reduces energy consumption by degrading non-essential components and provides nutrients needed for cell survival; in oxidative stress, autophagy eliminates damaged organelles (such as mitochondria) produced by ROS, alleviating oxidative damage (20). For example, hypoxia in the tumor microenvironment can induce autophagy, helping cancer cells tolerate nutrient deficiency and oxidative stress.

### 3.3 Participating in development and differentiation

Autophagy maintains the stemness and self-renewal ability of stem cells by removing damaged components (such as abnormal proteins and reactive oxygen species) in them. In muscle stem cells, autophagy deficiency leads to stem cell senescence, affecting muscle regeneration; activating autophagy can restore the regenerative function of muscle stem cells in aged mice (32). In addition, during embryonic development, autophagy is involved in cell differentiation and tissue remodeling, such as removing excess cells and organelles in the embryo to ensure tissue morphogenesis.

### 3.4 Cellular quality control

#### 3.4.1 Eliminating damaged or excess organelles and proteins

Autophagy removes dysfunctional organelles through selective degradation mechanisms (such as mitophagy and reticulophagy) to avoid their accumulation causing cellular damage. For example, Parkin- and PINK1-mediated mitophagy can clear damaged mitochondria to prevent excessive ROS production; autophagy can also remove β-amyloid aggregates related to Alzheimer’s disease (31). This selective elimination is the core defense line for cells against protein toxicity and organelle dysfunction.

#### 3.4.2 Maintaining genomic stability and inhibiting tumors

Autophagy reduces DNA damage by removing sources of ROS (such as damaged mitochondria) and participates in the turnover of DNA repair-related proteins to maintain genomic integrity. Deletion of autophagy genes such as Beclin 1 leads to genomic instability and increases the risk of tumorigenesis; autophagy can also promote cellular senescence by degrading nuclear membrane components (such as lamin B) to inhibit tumor progression (33). However, in the tumor microenvironment, autophagy may support the survival of tumor cells by providing nutrients, reflecting the duality of its functions.

### 3.5 Immune and inflammatory regulation

Autophagy participates in innate immune defense by eliminating intracellular pathogens (such as Mycobacterium tuberculosis and herpes virus), a process called “xenophagy”. For example, macrophages clear invading Salmonella through autophagy to limit its replication (31). In addition, autophagy can regulate inflammatory signaling pathways, inhibit excessive inflammatory responses by degrading inflammasome components (such as NLRP3); in adaptive immunity, autophagy participates in antigen presentation, promotes T cell activation, and enhances immune responses.

### 3.6 Affecting lifespan and health

Autophagic activity decreases with age, while enhancing autophagy can extend the lifespan of model organisms (nematodes, fruit flies, mice). For example, knocking out the Rubicon gene (an autophagy inhibitor) in mice can activate autophagy and improve aging-related phenotypes of the heart and kidneys; calorie restriction extends lifespan by inducing autophagy, and its effect depends on the function of autophagy genes (33). Defects in autophagy function are closely related to various age-related diseases (such as neurodegenerative diseases and metabolic syndrome), suggesting that it plays a core role in maintaining healthy aging.

## 4 Autophagy in emerging zoonotic infectious diseases

### 4.1 COVID-19

COVID-19 is a new viral respiratory infectious disease caused by Severe Acute Respiratory Syndrome Coronavirus 2 (SARS-CoV-2). The disease first broke out in Wuhan, China, and has currently caused a global pandemic, posing a great threat to public health and causing certain economic losses to society.

The symptoms described by the initial COVID-19 patients in Wuhan include fever, cough, difficulty breathing, headache, tachycardia, progressive hypoxemia, and other signs and symptoms. In addition, acute respiratory distress syndrome (ARDS), heart damage, kidney damage, multiple organ failure, and central nervous system damage are severe complications of COVID-19 that can lead to death. Currently, no effective targeted drugs have been developed, and only symptomatic treatment is provided for COVID-19 patients. Although vaccines have been developed, it remains an urgent task to find effective therapeutic targets and drugs. Exploring the mechanism of SARS-CoV-2’s invasion of cells can help researchers identify potential effective therapeutic targets.

SARS-CoV-2’s interaction with autophagy conforms to the “host protection-pathogen exploitation” dichotomy framework involving classical autophagy and autophagic flux disruption. Current studies are primarily based on Vero cells, A549 cells, primary human lung epithelial cells, and K18-hACE2 transgenic mouse models, with high consistency in core findings across different models. SARS-CoV-2 is an enveloped positive-sense single-stranded RNA virus (+single-stranded RNA, +ssRNA) and belongs to the Beta-coronavirus genus (34). Its main host is humans, and it is mainly transmitted through respiratory transmission, fecal-oral transmission, and indirect contact. This virus has a high degree of homology with bat coronaviruses. Although the possibility of infecting animals cannot be ruled out, there are currently no cases of animal infection. Spike (S) is one of the key structural proteins encoded by almost all coronaviruses, including SARS-CoV-2. The S glycoprotein consists of two subunits, namely the N-terminal S1 domain and the C-terminal S2 domain (35). The receptor-binding domain (RBD), which is responsible for recognizing the angiotensin-converting enzyme 2 (ACE2) receptor on target cells, is located on the S1 subunit. Meanwhile, the transmembrane domain, a functional element required for fusion, is located on the S2 subunit (36–38). The multi - basic S1/S2 protease cleavage site is an important feature of SARS - CoV - 2 virus entry (39). According to recent assessments, ACE2 and neuropilin-1 (NRP1) are key receptors for coronaviruses to enter host cells (40, 41). After the S protein binds to the ACE2 receptor, it needs to be primed before it can be further processed and cleaved by proteases in host cells, such as transmembrane protease serine 2 (TMPRSS2). This enables the fusion of the viral membrane with the host cell (42). After this fusion, SARS-CoV-2 enters the cell through endocytosis (endosomal and lysosomal pathways). Inside the cytoplasm, the uncoating process leads to the release of viral RNA. The genomic material of SARS-CoV-2 acts as messenger RNA (mRNA), and with the help of host ribosomes, it produces structural and non-structural proteins (Non-structural Protein, NSP). The 3’ end of the viral genome encodes four structural proteins, namely the spike protein (S), envelope protein (E), membrane protein (M), and nucleocapsid protein (N). The S protein is responsible for the attachment and fusion of the virus to host cell receptors. The N protein keeps the RNA gene stable within the envelope. The 5’ end of the RNA genome consists of two open reading frames (ORF); ORF1a and ORF1b, which encode 16 NSP (43). NSP is important component of viral RNA synthesis. The virus’s RNA-dependent RNA polymerase (RdRp) is a key enzyme of the replication/transcription complex (RTC), and it is formed by NSP in double-membrane vesicles (DMV) (44).

SARS-CoV-2’s entry and DMV-mediated replication process intersect with autophagic pathways, and its S protein, NSPs (e.g., NSP6, NSP13), and ORFs (e.g., ORF3a, ORF10) directly regulate autophagic innate immune signals, including the cGAS-STING and type I interferon pathways. This invasion-replication cascade has been validated in both immortalized cell lines and primary human lung cells, with consistent results supporting the critical role of autophagy modulation in viral propagation. For the replication of SARS-CoV-2, a full-length negative-sense genomic copy is initially created. Next, the above-mentioned copy serves as a template for the synthesis of new positive-sense genomic RNA. Finally, after the newly generated viral RNA and structural proteins are assembled in the endoplasmic reticulum and Golgi apparatus, new viral particles are released from the infected cells through the exocytosis of secretory vesicles (45, 46). Then, an increasing number of viruses spread downward to the alveoli and replicate within type II alveolar cells, leading to apoptosis and cell death (47, 48). At the same time, activated alveolar macrophages and lung epithelial cells release pro-inflammatory cytokines, triggering a cytokine storm and lung injury, leading to ARDS (49, 50).

Macroautophagy/autophagy is a conserved self - degradation process in cells activated under conditions including pathogen infection, cell starvation, and endoplasmic reticulum stress. It is the only pathway that can randomly or targetedly degrade entire organelles and invading pathogens (51). The autophagy process in COVID-19 can be divided into four stages: (1) initiation, (2) nucleation, (3) expansion, and (4) maturation/degradation. In the initial stage, after the invasion of SARS-CoV-2, the bioenergetic stress in host cells increases, thereby increasing the cytoplasmic AMP: ATP (Adenosine MonoPhosphate: Adenosine TriPhosphate) level. This leads to the Activation of adenosine 5’-monophosphate-activated protein kinase (AMPK) and the phosphorylation/inactivation of mammalian target of rapamycin 1(mTORC1).Then, in the nucleation stage, the ULK1 complex is activated, thereby activating the PtdIns3K complex and generating a region rich in PtdIns3P (PtdIns3P) on the surface of omegasomes. Then, PtdIns3P recruits WIPI and ZFYVE1 proteins that bind to omegasomes. In the expansion stage, autophagy-related proteins ATG7 and ATG3 work together to initiate Microtubule-Associated Protein 1 Light Chain3/Light Chain3 (MAP1LC3/LC3). The WIPI protein recruits binding factors, including complex of the Autophagy-related gene16-Like1 (ATG16L1), which leads to the binding of MAP1LC3/LC3 to phosphatidylethanolamine (generating LC3-II) to form autophagosomes. The substances encapsulated in autophagosomes will be degraded (52).

**Figure 3:**
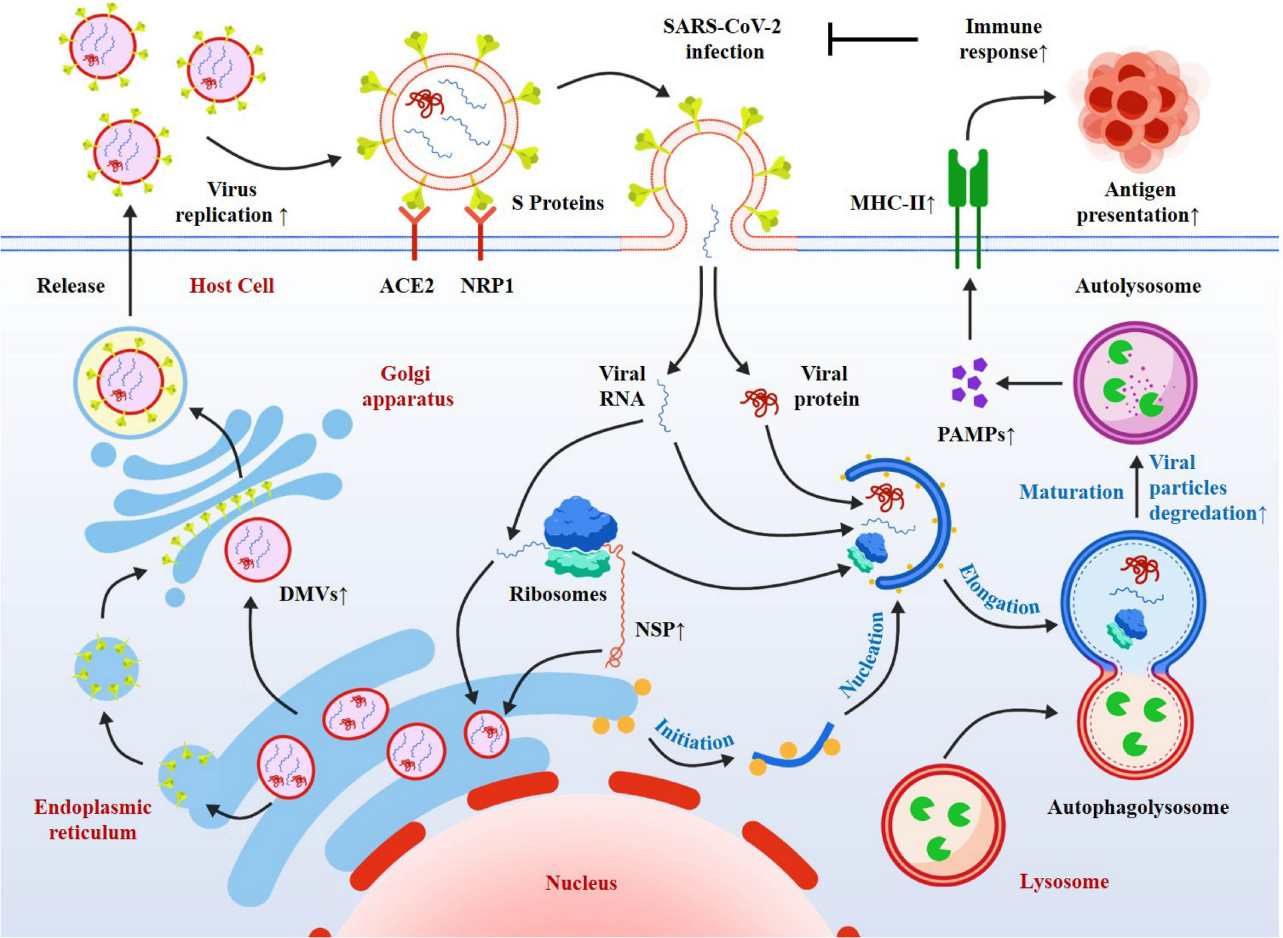
The Process of SARS-CoV-2 Infection and Its Interaction with Autophagy. The virus invades host cells by binding its S protein to the ACE2 and NRP1 receptors on the cell surface. Upon entry, it undergoes replication within the cells, generating viral RNA and proteins. Concurrently, the autophagy process is activated: autophagosomes encapsulate viral particles and subsequently fuse with lysosomes to form autolysosomes. Autolysosomes not only mediate the degradation of viral particles but also facilitate antigen presentation, thereby contributing to the initiation of an immune response. Meanwhile, the virus can also exploit relevant mechanisms to modulate processes like autophagy, so as to promote its own replication.

The autophagic response induced by SARS-CoV-2 is predominantly classical autophagy, regulated by the AMPK-mTORC1 and PI3K-AKT signaling axes, with stage-specific functional shifts reflecting autophagy’s type-specific regulatory features. Research controversies exist: some studies using Vero cells and A549 cells have reported that autophagy exerts a net antiviral effect via xenophagy, while others utilizing primary human bronchial epithelial cells and K18-hACE2 mice have emphasized the virus’s ability to hijack autophagy for replication. This discrepancy is primarily attributed to the infection stage (early antiviral vs. late pro-viral) and the inherent differences in autophagic regulatory networks between immortalized cell lines and primary cells. According to many studies, autophagy is a double-edged sword in SARS-CoV-2 infection. On the one hand, autophagy can directly degrade SARS-CoV-2 virus particles, promote the response to inflammatory cytokines, and facilitate antigen presentation to induce immunity against virus invasion. Both the SARS-CoV-2-derived protein ORF8 and the nucleocapsid protein (NP) can bind to La ribonucleoprotein 1(LARP1), and FKBP prolyl isomerase 7 (FKBP7), and inhibit mTORC1, thereby activating autophagy (53). It has been proven that during the infection of SARS-CoV-2, AMPK inhibits mTORC1 targets and selectively triggers Phosphatidylinositol 3-kinase/protein kinase B (PI3K/AKT1), thus leading to the initiation of autophagy and the encapsulation of viral particles. Then, the formation of autophagolysosomes is activated by these downstream signaling pathways, and then combines with lysosomes to eliminate their viral contents (54). On the other hand, SARS-CoV-2 manipulates autophagy to promote its survival and replication in host cells or evolves mechanisms to evade the host’s antiviral effects. The latest research shows that SARS-CoV-2 can inhibit complete autophagy and impede the maturation of autophagosomes in multiple ways, thereby blocking autophagic flux, including causing damage to mitophagy and resulting in mitochondrial dysfunction, etc. (55). (**Figure 3**) In the early stage of COVID-19, SARS-CoV-2 promotes its replication and evades the human body’s antiviral immune response by activating autophagy (56). After binding to the cell surface receptor ACE2, the SARS-CoV-2 enters the host cell through direct fusion with the plasma membrane (the cell surface pathway) or via endosomes/lysosomes (the endocytic pathway) (57, 58). Hoffmann et al. further demonstrated that TMEM41B is essential for SARS-CoV-2 infection,VMP1 and TMEM41B (two endoplasmic reticulum-resident transmembrane proteins) are crucial for the formation of double-membrane vesicles (DMV) during β-coronavirus infection (58, 59). Mohamud and colleagues reported that the overexpression of the papain-like protease of SARS-CoV-2 can cleave ULK1 and disrupt the formation of the ULK1-ATG13 complex, thereby blocking the host’s complete autophagy (60).Gassen and colleagues found that SARS-CoV-2 can limit autophagy signal transduction by activating the autophagy inhibitor S-phase kinase associated protein 2 (SKP2) and AKT1 and preventing the fusion of autophagolysosomes (61). Network analysis has shown that SARS-CoV-2 can block autophagic flux by negatively regulating autophagy genes, synaptosome associated protein 29 (SNAP29), and lysosomal acidification genes in infected human nasopharyngeal samples, thereby reducing the degradation of viral particles (62). Miao and colleagues have shown that the ORF3a of SARS-CoV-2 can dissociate homotypic fusion and protein sorting components, thereby inhibiting the fusion of autophagosomes and lysosomes (63). Bouhaddou and colleagues found that the AMPK-MAPK14/p38 pathway was activated while the AKT pathway was inhibited, which could induce autophagy in COVID-19. Incomplete autophagy was caused by the interference of the virus in double-membrane vesicles (DMV) with the fusion of autophagosomes and lysosomes under such circumstances, and further promoted viral replication (64). Meanwhile, SARS-CoV-2 has evolved multiple components and mechanisms to evade the host’s antiviral effects. After being infected by SARS-CoV-2, mitophagy is activated through the Pink1/Parkin (PTEN-induced putative kinase 1/E3 ubiquitin-protein ligase parkin) pathway. SARS-CoV-2 inhibits mitophagy by suppressing the binding of autophagy proteins P62 and LC3 (54). The SARS-CoV-2-derived ORF3a can inhibit mitochondrial fusion by binding to the host proteins Mitofusin (Mfn) 1 and 2 (52). The research by Madeddu et al. showed that SARS-CoV-2 ORF-9b causes mitochondrial stress and induces its fusion and elongation. Meanwhile, it promotes autophagy and hinders mitophagy, thus enabling the survival of the infected cells (65). Gassen et al. showed that SARS-CoV-2 can inhibit autophagic flux by interfering with multiple metabolic pathways, including inhibiting glycolysis by suppressing AMPK and mTORC1 (66). Research by Chao Sui, Xiao Tongyang and others has shown that NSP13 inhibits the production of type I interferon by recruiting TBK1 (TANK binding kinase 1) to p62 for autophagic degradation, enabling it to evade the host’s innate immune response (67); Li Xingyu and others found that ORF10 degrades MAVS (mitochondrial antiviral signaling protein) through mitophagy to suppress the antiviral innate immune response (68). The overexpression of ORF10 inhibits the phosphorylation, translocation of interferon regulatory factor 3 induced by cGAS–STING and the subsequent interferon induction. Mechanistically, ORF10 interacts with STING, weakens the STING-TBK1 association, and impairs STING oligomerization, aggregation as well as STING-mediated autophagy. It has also been confirmed that ORF3a can inhibit cGAS-STING-mediated autophagic flux and antiviral functions (69); A significant correlation between the uncontrolled inflammation triggered by SARS-CoV-2 and autophagy defects has been verified (as reported by Garcia-Perez et al. in 2020). This indicates that the occurrence of the enhanced cytokine storm might stem from the failure of autophagy in maintaining homeostatic regulation (70). However, in the late stage of COVID-19, autophagy acts as a negative regulator of the inflammatory response. Studies by some researchers have shown that in the late stage of SARS-CoV-2 infection, the increased viral levels can disrupt the structure and function of mitochondria, leading to elevated levels of reactive oxygen species. Subsequently, the NLRP3 inflammasome (NOD-, LRR- and pyrin domain-containing protein 3) is activated and pro-inflammatory cytokines such as IL-1β and IL-18 are produced. At this stage, autophagy can clear damaged mitochondria and reactive oxygen species, and degrade the NLRP3 inflammasome and its key components, including NLRP3, PYD and CARD domains (PYCARD/ASC), and caspase-1 (CASP-1) (71).

The dual role of autophagy in SARS-CoV-2 infection reflects the evolutionary interplay between host xenophagic clearance and viral autophagy hijacking. Consistent across cell models, animal models, and clinical samples, autophagy functions as an antiviral mechanism in the early infection phase and a pro-inflammatory regulator in the late phase. An unresolved controversy is whether targeted modulation of autophagic flux can simultaneously mitigate viral replication and cytokine storm, as current evidence supports stage-specific therapeutic strategies. Precisely due to the above-mentioned reasons, autophagy has emerged as a promising target in the fight against and treatment of COVID-19. Bestion et al. adopted the K18-hACE2 mouse model and demonstrated that GNS561 displays robust antiviral activity against SARS-CoV-2 by means of autophagy inhibition (72); There are also studies showing that inhibiting the SUMOylation of ACE2 can prevent SARS-CoV-2 infection through TOLLIP-mediated selective autophagy (73); Zhang Yabin and others found that the main protease NSP5 of SARS-CoV-2 cleaves the selective autophagy receptor Sequestosome 1 (SQSTM1/p62), which can prevent the autophagic degradation of viral membrane proteins (74). Furthermore, Gilani et al. revealed that vitamin D is capable of negatively regulating the key targets within the mTOR pathway, which serves to inhibit autophagy. Specifically, vitamin D promotes the production of type I interferon. Concurrently, by elevating the intracellular calcium levels, vitamin D stimulates the activity of enzymes crucial for autophagy. This includes class III phosphatidylinositol 3 - kinase (PI3KC3) and the autophagy - associated protein Beclin 1 encoded by autophagy - related genes (75). Given the increasing number of SARS-CoV-2 cases and the waning efficacy of vaccination - induced immunity, research on drugs capable of hindering the transmission of the SARS-CoV-2 virus has become of utmost importance. Rapamycin, for instance, is a medication that specifically targets the mTOR signaling system. In laboratory animal studies, rapamycin, in combination with other autophagy - inducing agents like metformin, statins, and carbamazepine, has been demonstrated to exhibit antiviral characteristics (76); The combined application of wheat germ spermidine and clove eugenol has the ability to stimulate autophagy *in vitro*. This combinatorial treatment exhibits the potential to bolster the immune system’s defense against infections. Intriguingly, recent studies have demonstrated that wheat germ spermidine can induce autophagy through directly suppressing mTORC1 and upregulating the expression of SQSTM1/p62 (77). During the initial phase of the pandemic, numerous scientific studies suggested that chloroquine/hydroxychloroquine could be highly efficacious in treating severe COVID -19 patients. Nevertheless, given that chloroquine/hydroxychloroquine permanently inhibits autophagy, it exerts significant adverse effects on cells (78). A remarkable feature of these potential SARS-CoV-2 therapies is that they target multiple host-virus interactions, particularly the mTOR pathway, which is crucial for the virus’s replication ability (45). Accumulating evidence from recent research suggests that three novel oral antiviral agents, namely molnupiravir, fluvoxamine, and Paxlovid, hold promise in effectively decreasing the mortality and hospitalization rates among COVID - 19 patients. Significantly, these medications have not been associated with an elevated incidence of adverse events, demonstrating favorable overall safety profiles. Currently, these three oral antiviral drugs remain under active investigation. Available data imply that they may offer new prospects for the convalescence of COVID - 19 patients and potentially represent breakthrough therapeutic modalities for the treatment of COVID - 19 (79).

Based on the stage-specific dual role model of autophagy, targeting early autophagic initiation (e.g., mTOR activation) or late autophagic flux (e.g., autophagosome-lysosome fusion) represents a promising therapeutic strategy for COVID-19. Autophagy-modulating agents such as GNS561 and rapamycin combinations have been validated in K18-hACE2 mouse models, showing reduced viral load and alleviated lung injury, but require further confirmation in large-scale clinical trials to assess long-term safety and efficacy across diverse patient populations.

### 4.2 SARS and MERS

Before the COVID-19 pandemic, we witnessed the emergence of two highly pathogenic zoonotic diseases in succession: Severe Acute Respiratory Syndrome (SARS) and Middle East Respiratory Syndrome (MERS-CoV). They are caused by Severe Acute Respiratory Syndrome Coronavirus (SARS-CoV) and Middle East Respiratory Syndrome Coronavirus (MERS-CoV) respectively. Both of these viruses belong to the β-coronavirus genus. After being infected with SARS-CoV, patients may experience flu-like symptoms and pneumonia. In severe cases, it can lead to fatal respiratory failure and acute respiratory distress syndrome (80); Infection with MERS - Cov can cause hosts to develop symptoms such as fever, headache, and fatigue, ultimately leading to lung and kidney failure (81, 82); These two viruses pose a significant threat to human health.

Both SARS-CoV and MERS-CoV follow the “host protection-pathogen exploitation” dichotomy framework in their interactions with autophagy, involving classical autophagy and autophagic flux modulation. Current studies on SARS-CoV are primarily based on Vero cells, rhesus macaque models, and primary human respiratory epithelial cells, with high consistency in core findings; MERS-CoV research relies on Vero cells, dromedary camel models, and human lung cell lines, though *in vivo* data on autophagy modulation remains relatively limited compared to SARS-CoV. The genomic characteristics and viral replication processes of SARS - CoV and MERS - CoV can be referred to the descriptions of SARS - CoV - 2 in the above - mentioned content. When it comes to their interactions with autophagy, due to variances in viral components, SARS - CoV and MERS - CoV display distinct behaviors.

The entry and DMV-mediated replication processes of both viruses intersect with autophagic pathways, where their NSPs (e.g., NSP3, NSP6) and PLpro domains directly regulate autophagic innate immune signals, including STING-mediated type I interferon production. This pathway crosstalk has been validated in multiple cell models for SARS-CoV, while MERS-CoV’s autophagy-immune interactions require further confirmation in primary human cells to align with *in vitro* findings. Analogous to other RNA viruses, coronaviruses (primarily their non - structural proteins (NSPs) and open reading frames (ORFs)) engage in interactions with the cellular autophagy pathway, which facilitates their replication (83, 84). The development of double - membrane vesicles originating from the endoplasmic reticulum within the host cytoplasm bears a striking resemblance to that of autophagosomes. This similarity suggests that coronaviruses are emulating the cellular autophagy pathway. In the context of numerous RNA viruses, these double - membrane vesicles function as genomic replication location. Substantial evidence has demonstrated that virus - induced double - membrane vesicles (DMVs) serve as the sites for coronavirus RNA synthesis (85). The double - membrane vesicles (DMVs) induced by the virus bear a strong resemblance to autophagosomes and are likely to fuse with late endosomes or lysosomes.

For SARS-CoV, the induced autophagic response is classical, regulated by BECN1 and mTOR signaling, while MERS-CoV exhibits dual regulation of autophagy (initiation promotion and flux inhibition) via AKT1-SKP2 and SNARE complex pathways. Research controversies exist for SARS-CoV: early Vero cell studies proposed that PLpro-induced autophagy is fully pro-viral, while recent primary respiratory epithelial cell studies suggest a partial antiviral role of autophagy in limiting viral spread. For MERS-CoV, conflicting reports exist regarding BECN1 regulation-some Vero cell studies show BECN1 ubiquitination and autophagy attenuation, while others indicate PLpro-mediated BECN1 stabilization-likely due to differences in viral strain and infection dosage. Accumulating evidence indicates that the expression of nsp3 and nsp4 in both SARS - CoV and MERS - CoV prompts the formation of DMVs. These DMVs play a crucial facilitating role in the DMV - endosome fusion step within the canonical autophagy pathway (86, 87). SARS-CoV infection serves as a powerful activator for the formation and accumulation of autophagosomes. It is likely that SARS-CoV infection induces autophagy through specifically downregulating the expression of ACE2, which acts as the viral entry receptor (88–90). Several SARS-CoV proteins have been demonstrated to induce autophagy via diverse mechanisms *in vitro*. Cottam et al. have provided evidence that the nsp6 protein of the coronavirus initiates autophagy from the endoplasmic reticulum through the omegasome intermediate and also restricts the expansion of autophagosomes (91, 92). Moreover, the papain-like protease (PLpro) encoded by the SARS-CoV ORF1a is capable of interacting with the autophagy regulator BECN1 and performing deubiquitination on it. Through this process, BECN1 can be protected from degradation, thereby prolonging its stability (89, 90, 93). Notably, the intracellular accumulation of SARS-CoV ORF8b initiates cellular stress through the activation of the transcription factor EB as well as its target genes, which consequently results in the augmentation of autophagic flux (94). Moreover, Chen et al. have also provided evidence that the transmembrane-associated papain-like protease (PLpro-TM) can induce incomplete autophagy. Specifically, it achieves this by impeding autophagosome maturation and inhibiting the fusion between autophagosomes and lysosomes, which consequently leads to the accumulation of a substantial number of autophagosomes within cells (95).

The dual role of autophagy in SARS-CoV infection reflects the balance between host xenophagic clearance and viral autophagy hijacking, consistent across cell and animal models. For MERS-CoV, the autophagic role remains less defined due to conflicting model data, but current evidence supports a predominantly pro-viral function via autophagic flux blockage. An unresolved controversy common to both viruses is whether autophagy modulation can synergize with antiviral therapies, as preclinical data is limited to single-agent studies. MERS-CoV infection has been shown to block the fusion of autophagosomes and lysosomes by interfering with the interaction of the SNARE complex proteins STX17-VAMP8-SNAP29, which is mainly mediated by NSP6. Moreover, MERS-CoV activates SKP2 by increasing the phosphorylation of AKT1, which causes the ubiquitination of BECN1 and consequently leads to the attenuation of autophagy (96). However, similar to the corresponding SARS-CoV NSP3, MERS-CoV NSP3 possesses a papain-like protease (PLpro) domain, which interacts with BECN1 and performs deubiquitination to increase its abundance (88, 89, 94), which is conducive to the initiation of autophagy. However, at present, the exact role of autophagy in MERS-CoV infection remains unclear. Multiple studies have already shown that enhancing autophagy might be a new therapeutic target for controlling MERS-CoV infection (95, 96). In terms of the role of autophagy in virus replication, previous studies have demonstrated that the PLpro-TM of either SARS-CoV or MERS-CoV might directly interact with BECN1, which could then sequester the crucial innate immune signaling adaptor STING into autophagosomes. Such a process would impede the downstream antiviral response and consequently promote virus replication (94). However, due to the complexity of the autophagy pathway and its mechanism of action, there is still ample room for research regarding the role of autophagy in the infection and replication of SARS-CoV and MERS-CoV.

Based on the autophagy-pathogen interaction models, targeting SARS-CoV’s PLpro or MERS-CoV’s NSP6/SKP2 represents promising therapeutic directions. For SARS-CoV, PLpro inhibitors have shown efficacy in rhesus macaque models by restoring autophagic flux and reducing viral load; for MERS-CoV, SKP2 inhibitors have demonstrated potential in Vero cell and camel models by enhancing autophagy-mediated viral clearance. Both strategies require further validation in human clinical settings to assess safety and cross-strain efficacy.

### 4.3 Ebola hemorrhagic fever

Ebola hemorrhagic fever is a virulent infectious disease that is shared by humans and non-human primates and is induced by the Ebola virus. The Ebola virus (abbreviated as Ebov) is a filamentous, enveloped RNA virus, which exploits the autophagic endosome pathway to achieve cell infection (97), it relies on its structural protein (Glycoprotein, GP) to invade the host. The viral protein 40 (VP40) is responsible for the assembly and budding of the virus (98).

EBOV’s interaction with autophagy aligns with the “host protection-pathogen exploitation” dichotomy framework, primarily involving xenophagy (a subset of classical autophagy) and ER stress-mediated autophagy. Current studies are based on Vero cells, primary human macrophages, and cynomolgus monkey models, with moderate consistency in autophagy-related findings across models—cell line data is more abundant, while *in vivo* validation of autophagic mechanisms remains limited. The autophagy triggered by activating the pathways related to endoplasmic reticulum (ER) stress contributes to the host’s immune response to EBOV infection (99, 100).

EBOV’s macropinocytosis-mediated entry and VP40-driven budding intersect with autophagic pathways, where viral GP and host BECN1/LC3B directly regulate autophagic innate immune signals, including type I interferon production and inflammasome activation. This pathway crosstalk has been validated in Vero cells and primary macrophages, though the intensity of autophagic induction varies between immortalized and primary cell models. Numerous studies have demonstrated that the Ebola virus enters host cells via macropinocytosis. Moreover, the internalization process of the Ebola virus is under the control of autophagy-related proteins. In cells deficient in the recombinant protein of Beclin 1 (BECN1), the autophagy-related gene 7 (Atg7), or the light chain 3 beta (LC3B) of the autophagy microtubule-associated protein, the internalization of vesicles on the cell surface is significantly inhibited. Nevertheless, these autophagy-related proteins do not exert an impact on the formation of ebov-ankfy1 on the cell surface. Consequently, it is further verified that the uptake of the Ebola virus takes place on the cell membrane, and autophagy-related proteins are likely to govern the early steps of EBOV uptake in the vicinity of the cell surface (101).

The autophagic response induced by EBOV is predominantly classical xenophagy, regulated by the ER stress-ATF6/IRE1α and BECN1-LC3B signaling axes. Research controversies exist: Vero cell studies report that autophagy primarily exerts an antiviral effect via GP degradation, while cynomolgus monkey models suggest a dual role—early xenophagy restricts viral entry, but late-stage autophagy is hijacked to promote VP40 budding. This discrepancy may stem from the dynamic interplay between viral replication stages and host immune responses in complex *in vivo* environments. Furthermore, protein disulfide isomerase A3 (PDIA3) can inhibit the expression of the Ebola virus glycoprotein (GP) in the endoplasmic reticulum (ER) through the autophagy-lysosome pathway (102). The endoplasmic reticulum-selective autophagy receptor FAM134B can inhibit the replication of Ebola strains Mayinga and Mokona (103); Multiple studies have shown that the reproduction of viruses requires chaperone proteins, and AR-12 (OSU-03012) can inhibit the functions of chaperone proteins and stimulate the formation of autophagosomes, thereby exerting an antiviral effect (104); It has been verified that the molecular chaperone protein BAG3 (B cell lymphoma 2-associated anthanogene 3) plays a negative regulatory role in the budding of Ebola virus VP40 virus-like particles (VLPs) as well as infectious viruses (105).

The dual role of autophagy in EBOV infection reflects the evolutionary interplay between host xenophagic clearance and viral exploitation of autophagic machinery. Consistent across cell and animal models, autophagy functions as an antiviral barrier in early infection, while its late-stage hijacking facilitates viral egress. An unresolved controversy is whether targeted modulation of autophagic timing (e.g., enhancing early xenophagy while inhibiting late autophagic budding) can optimize therapeutic outcomes.

Based on the xenophagy-centered interaction model, targeting ER stress-mediated autophagy activation (e.g., FAM134B agonists) or chaperone-dependent autophagic hijacking (e.g., BAG3 inhibitors) represents promising therapeutic directions. Agents such as AR-12 have demonstrated antiviral efficacy in Vero cells and ex vivo human tissue models by inducing protective autophagy, but require further validation in non-human primate models and clinical trials to assess *in vivo* safety and efficacy against circulating EBOV strains.

### 4.4 Dengue fever

Dengue fever is a mosquito-borne viral disease (classified as an arbovirus) that is induced by the Dengue virus (abbreviated as DENV). DENV pertains to the Flaviviridae family of viruses and encompasses primarily four serotypes (namely DENV1 - DENV4). Infection with DENV may give rise to diseases presenting a spectrum of severe symptoms, varying from mild dengue fever to life-threatening conditions such as Dengue Shock Syndrome (DSS) and Dengue Hemorrhagic Fever (DHF) (106, 107). DHF represents a severe form of dengue fever. Typically, it is triggered by homologous reinfection or secondary infection involving different serotypes of the Dengue virus (DENV). The condition is marked by thrombocytopenia, liver injury, and bleeding manifestations. Moreover, DHF can ultimately give rise to DSS, which poses a significant threat as it may result in mortality (108, 109). Furthermore, the antibody-dependent enhancement (ADE) of the DENV can lead to an increase in the virus’s pathogenicity and virulence. Its mechanism is rather complex and awaits further in-depth research (110, 111). To date, effective antiviral drugs for curing Dengue virus (DENV) infections and vaccinations against DENV infections are still unavailable and inadequately developed (112).

DENV’s interaction with autophagy conforms to the “host protection-pathogen exploitation” dichotomy framework, involving classical autophagy and ER stress-mediated autophagic responses. Current studies are primarily based on Huh7 cells, Vero cells, primary human hepatocytes, and mouse models, with moderate consistency across models—most *in vitro* studies support a pro-viral role of autophagy, while *in vivo* models reveal a more balanced dual function. It has been reported that Dengue virus (DENV) infection induces autophagy, and this process is mediated by the high-mobility group box 1 protein (HMGB1) (113).

DENV’s TIM-1-mediated entry and ER-targeted replication processes intersect with autophagic pathways, where viral NS4A/NS1 proteins and host HMGB1 coregulate autophagic innate immune signals, including NF-κB and type I interferon pathways. This crosstalk has been validated in multiple cell models, though ADE conditions may alter autophagic regulation intensity compared to primary infection. Autophagy exerts a promoting effect on virus replication, and there are three potential pathways implicated, specifically the AMPK pathway, the endoplasmic reticulum (ER) stress pathway, and the ataxia telangiectasia mutated (ATM) kinase pathway. Moreover, the non-structural proteins NS4A and NS1 of the Dengue virus (DENV) are capable of inducing the activation of autophagy as well. The Dengue virus targets the endoplasmic reticulum (ER) and induces stress therein. During the life cycle of the virus, the accumulation of unfolded proteins within the ER triggers three host unfolded protein response (UPR) pathways, specifically inositol-requiring protein-1α (IRE1α), protein kinase RNA-like endoplasmic reticulum kinase (PERK), and activating transcription factor-6 (ATF6), all of which subsequently activate autophagy (114, 115); Both the AMPK and ATM pathways activate autophagy by inhibiting the activity of mTOR (115). It is worth noting that studies have shown that T cell/transmembrane immunoglobulin and mucin domain-containing protein-1 (TIM-1), a type I transmembrane glycoprotein existing in multiple viruses and also regarded as a cell receptor that promotes viral infection (116), is the receptor through which the Dengue virus (DENV) enters cells (117). TIM-1 exhibits a dual role of promoting viral entry and activating autophagy during DENV infection (118).

The autophagic response induced by DENV is classical, regulated by the AMPK-mTOR and ER stress-UPR signaling axes. Research controversies exist: Huh7 and Vero cell studies consistently report autophagy as pro-viral via glycolysis enhancement, while primary hepatocyte and mouse model studies reveal an antiviral role of p62-mediated capsid protein degradation. This discrepancy stems from cell type-specific autophagic cargo selectivity—immortalized cell lines prioritize autophagy-mediated metabolic support for virus replication, whereas primary cells activate selective xenophagy for viral clearance. Autophagy induced by the Dengue virus (DENV) exerts an impact on DENV replication through diverse mechanisms. DENV infection necessitates glycolysis to attain optimal replication efficiency (119). Li Yingrui and colleagues have provided evidence that enhancing autophagic activity in DENV-infected cells can further augment cellular glucose uptake, glucose transport, glycolytic processes, and viral titers. Intriguingly, their findings also revealed that inhibiting glucose uptake led to a reduction in viral replication, while paradoxically increasing autophagic activity (120). Studies conducted by Wu Yaoxing et al. have illustrated that the capsid protein of the Dengue virus serves as a key viral protein in the processes of virus assembly, maturation as well as replication. The autophagy cargo receptor p62 transports the ubiquitinated capsid protein to autophagosomes for degradation, which suggests the potential role of p62 in constraining the replication of the Dengue virus (121).

The dual role of autophagy in DENV infection reflects the interplay between host selective xenophagy and viral exploitation of autophagy for metabolic support. Consistent across models, autophagy’s function is context-dependent—pro-viral in early replication and antiviral in late infection phases. An unresolved controversy is how ADE modulates autophagic responses, as current data primarily focuses on primary DENV infection. All of the aforementioned studies offer valuable references for the exploration of the pathogenesis as well as the treatment approaches regarding Dengue virus infection. Autophagy holds the potential to emerge as a promising therapeutic target for dengue fever.

Based on the context-dependent autophagy model, targeting DENV-induced ER stress or AMPK-mTOR signaling represents a promising therapeutic direction. Autophagy inhibitors targeting the AMPK pathway have shown efficacy in Huh7 cell and mouse models by reducing viral load, while strategies enhancing p62-mediated selective autophagy may complement antiviral effects in primary cells. These approaches require further validation in clinical settings, particularly under ADE conditions, to assess safety and serotype-independent efficacy.

### 4.5 AIDS

Acquired Immune Deficiency Syndrome (AIDS) is an immune system deficiency disease caused by the infection of Human Immunodeficiency Virus (HIV). After being infected with HIV, patients may develop a series of opportunistic infections and malignant tumors, and in severe cases, it can lead to death. HIV belongs to the Retroviridae family and is an enveloped virus, which is mainly transmitted through body fluids and vertical mother-to-child transmission. HIV1 and HIV2 are its two main subtypes, and most outbreaks involve HIV1. HIV has two main glycoproteins, GP120 and GP41, which are essential for the virus to attach to and enter cells. The two most common co-receptors are CCR5 and CXCR4. CD4+ T lymphocytes are the main targets of this virus. During the process of virus entry, GP120 recognizes and binds to CD4 molecules, while GP41 recognizes and binds to cell co-receptors (122). The clinical latency period of HIV infection can extend up to 10 years. In the advanced stage of infection, a sharp elevation in viral load and a significant decline in the number of CD4+ T lymphocytes occur within the patient’s body, thereby resulting in the development of AIDS. Owing to the high level of mutations present within the viral genome, an effective vaccine remains to be developed (123).

HIV’s interaction with autophagy adheres to the “host protection-pathogen exploitation” dichotomy framework, featuring cell type-specific modulation of classical autophagy and xenophagy. Current studies are primarily based on CD4+ T lymphocytes, macrophages, dendritic cells, and SCID mouse models, with high consistency in core findings—HIV exhibits dual regulation of autophagy (induction and suppression) across different cell types, though the underlying mechanisms vary. It is worth noting that HIV has been verified to possess the ability to both induce and suppress autophagy. During the early stages of viral infection, HIV infection triggers autophagy while simultaneously blocking it to evade viral clearance.

HIV’s GP120/CD4-mediated entry and intracellular replication processes intersect with autophagic pathways, where viral accessory proteins (Tat, Nef, Env) and host factors (IRGM, BECN1, LAMP2A) coregulate autophagic innate immune signals, including type I interferon production and inflammatory cytokine release. This pathway crosstalk has been validated in multiple cell models, with consistent demonstration of HIV’s ability to manipulate autophagy for immune evasion. Specifically, HIV Tat can result in the impairment of autophagy within macrophages. Moreover, Tat inhibits neuronal autophagy and restricts the fusion of autophagosomes with lysosomes through its interaction with LAMP2A. This might explain the occurrence of neuronal apoptosis as a consequence of the inhibited autophagy in the context of HIV-associated neurocognitive disorders (HAND). Additionally, Tat induces autophagy by interacting with BAG3 in glial cells (124). Nef, which is an accessory protein of HIV-1, interacts with IRGM. Subsequently, IRGM is capable of activating autophagy by promoting the assembly of the ULK1/BECLIN-1/ATG16 complex. Castro-Gonzale et al. have conducted research and demonstrated that HIV-1 Nef counteracts the restriction on autophagy by enhancing the connection between BECN1 and its inhibitor BCL2 in a PRKN-dependent manner (125). Nef, which is an accessory protein of HIV-1, interacts with IRGM. Subsequently, IRGM is capable of activating autophagy by promoting the assembly of the ULK1/BECLIN-1/ATG16 complex.

The autophagic response modulated by HIV is classical, with regulation involving the mTOR, ULK1/BECN1, and LAMP2A signaling axes. Research controversies exist: some macrophage studies report that autophagy inhibition restricts viral replication, while others suggest that Nef-induced autophagy facilitates viral persistence. This discrepancy is attributed to the activation stage of autophagy (early induction vs. late flux blockage) and the polarization state of macrophages (M1 vs. M2). In dendritic cells, consistent findings support Env-mediated mTOR activation and autophagy suppression for efficient viral production, with no major conflicting reports. In macrophages, both infected and uninfected macrophages can eliminate viral replication through autophagy inhibition (126); In dendritic cells, HIV-1 Env activates the m-TOR pathway and induces the rapid shutdown of autophagy in dendritic cells, thus achieving efficient viral replication and virus production (127–130); In the central nervous system, HIV-1 Env may trigger HIV-associated neurocognitive disorders (HAND) by inducing autophagy (131).

**Figure 4:**
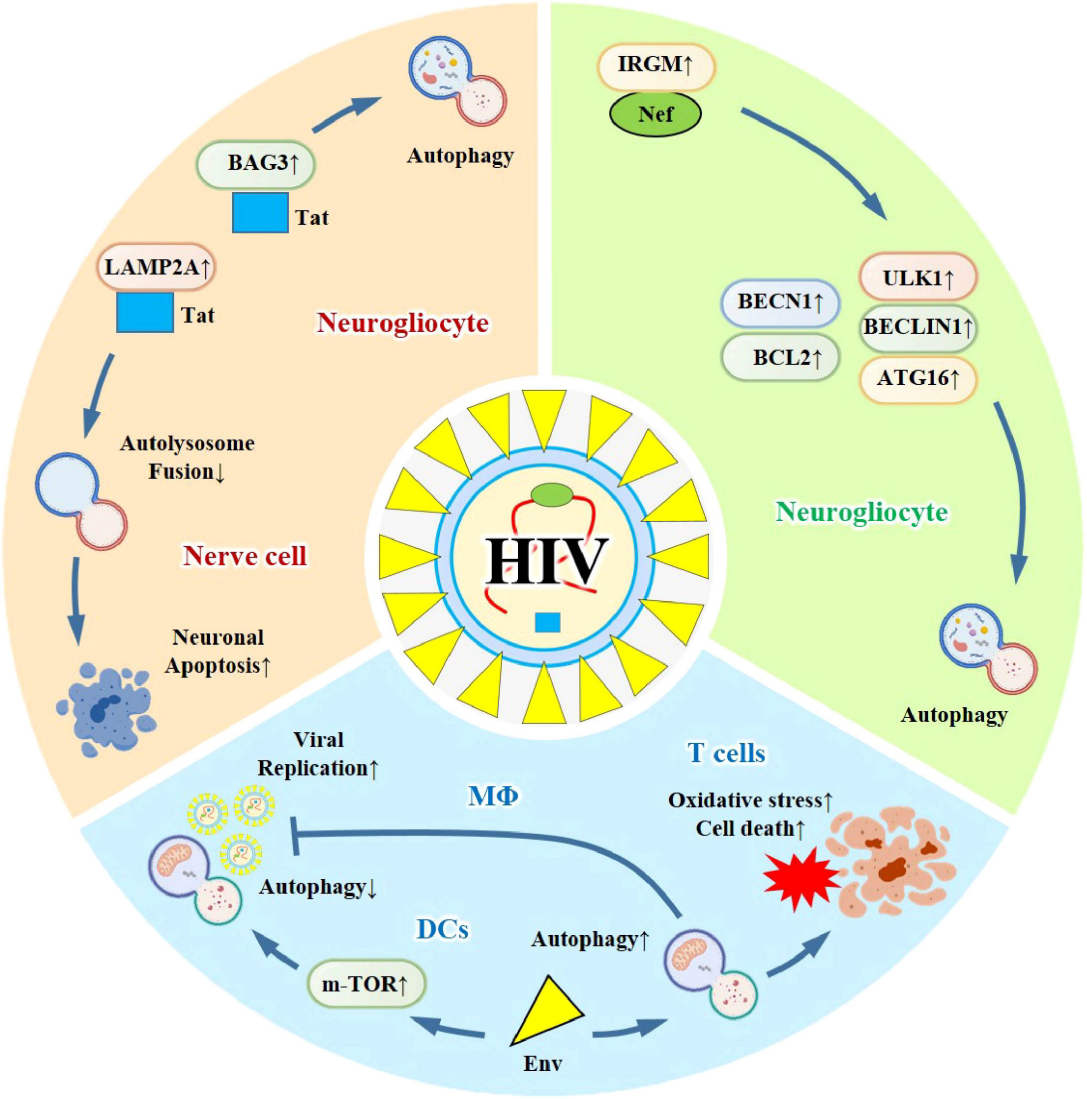
HIV - autophagy interaction in diverse cell types and its therapeutic potential. HIV exhibits dual regulatory effects on autophagy: within neurogliocytes, it induces autophagy through Tat and Nef; in nerve cells, Tat impairs the fusion of autolysosomes, thereby triggering apoptosis; in T cells, it results in oxidative stress and subsequent cell death; in macrophages, autophagy is inhibited to facilitate viral replication; and in dendritic cells, the Env protein of HIV activates the m-TOR pathway, which in turn suppresses autophagy to enable efficient viral replication. Investigating this interaction holds promise as a potential therapeutic target for HIV infection.

The dual role of autophagy in HIV infection reflects the evolutionary interplay between host xenophagic clearance (e.g., in macrophages) and viral exploitation of autophagy for immune evasion and replication (e.g., in dendritic cells and glial cells). Consistent across cell and animal models, HIV’s ability to selectively modulate autophagy in a cell type-specific manner is a key pathogenic feature. An unresolved controversy is whether autophagy modulation can eliminate HIV latent reservoirs, as current evidence focuses on active viral replication rather than latency. A large number of experimental studies have shown that HIV-mediated autophagy plays a very important role in the process of viral infection (132). In conclusion, studying the interaction between autophagy and HIV infection can become a potential therapeutic target for controlling HIV infection.(**Figure 4**).

Based on the cell type-specific autophagy modulation model, targeting HIV accessory proteins (Tat, Nef) or host autophagic regulators (mTOR, BECN1) represents a promising therapeutic direction. Autophagy inducers such as rapamycin have shown efficacy in SCID mouse models by enhancing viral clearance in macrophages, while strategies inhibiting Nef-mediated autophagy manipulation may restore antiviral immunity in dendritic cells. These approaches require further validation in clinical trials to assess safety and potential synergy with antiretroviral therapy (ART).

### 4.6 Rotavirus disease

Rotavirus is a non-enveloped double-stranded RNA virus that often causes viral diarrhea in infants and can lead to the deaths of hundreds of thousands of infants every year (133).

Rotavirus’s interaction with autophagy aligns with the “host protection-pathogen exploitation” dichotomy framework, primarily involving classical autophagy activated via calcium-mediated signaling. Current studies are predominantly based on MA104 cells, primary intestinal epithelial cells, and suckling mouse models, with high consistency across models—autophagy is uniformly reported to promote viral replication, though minor differences exist in signaling pathway activation intensity between cell lines and primary cells. Upon the invasion of the host organism by rotavirus, the autophagy of host cells is activated through the calcium ion-mediated signaling pathway. Rotavirus non-structural protein 4 (NSP4), which is an endoplasmic reticulum transmembrane glycoprotein possessing a viroporin domain (VPD), has the ability to elevate the level of calcium ions within mammalian cells (134).

Rotavirus’s NSP4-mediated calcium release and VP7-driven assembly processes intersect with autophagic pathways, where viral NSP4, vsRNA1755, and host CAMKK2/AMPK coregulate autophagic signaling, with no direct involvement in innate immune signal modulation reported to date. This pathway crosstalk has been validated in both MA104 cells and primary intestinal epithelial cells, confirming consistent autophagy activation for viral benefit. Rotavirus encodes the viral porin NSP4, through which calcium ions stored in the endoplasmic reticulum of host cells are released into the cytoplasm. The elevation of cytoplasmic calcium ion concentration then activates calcium/calmodulin-dependent protein kinase 2 (CAMKK2). Once activated, CAMKK2 phosphorylates 5’-adenosine monophosphate-activated protein kinase (AMPK). Subsequently, this phosphorylation event leads to either the inhibition of mTOR complex 1 (mTORC1) or the direct phosphorylation of Unc-51 like autophagy activating kinase 1 (ULK1), thereby triggering the initiation of autophagy (135, 136).

The autophagic response induced by rotavirus is classical, regulated by the calcium-CAMKK2-AMPK-mTORC1 and PI3K/Akt/mTOR signaling axes. Research controversies are minimal, though a few primary cell studies suggest a weak antiviral role of autophagy in limiting excessive viral load, which contrasts with the strong pro-viral evidence from MA104 cells and suckling mouse models. This minor discrepancy is attributed to the more robust innate immune response in primary intestinal epithelial cells compared to immortalized cell lines. Rotavirus exerts an impact on the transport of autophagic membranes. Specifically, it takes advantage of the autophagic membrane transport pathway to translocate the viral protein VP7 from the endoplasmic reticulum to the viroplasm. This process facilitates the assembly of double-layered virus particles (DLP) with VP7, resulting in the formation of infectious VLP virus particles, which in turn is beneficial to the replication of rotavirus (137–139).

The pro-viral role of autophagy in rotavirus infection reflects the virus’s successful exploitation of host autophagic pathways for membrane transport and replication niche formation, consistent across all major experimental models. An unresolved question is whether targeting autophagy can mitigate severe diarrhea in clinical settings, as preclinical data is limited to viral load reduction rather than pathological symptom improvement. After rotavirus infection, it will lead to the activation of the classic regulatory autophagy signaling pathway of class I phosphatidylinositol-3-kinase/protein kinase B/the mammalian target of rapamycin (PI3K/Akt/mTOR). Rotavirus NSP4 encodes vsRNA1755. In the early stage of infection, vsRNA1755 targets the insulin-like growth factor 1 receptor (IGFI R) of host cells, inhibits the PI3K/Akt/mTOR pathway and activates autophagy, which is beneficial to the reproduction of the virus itself (140–144). In the late stage of infection, microRNA let-7g is downregulated, which promotes the inhibition of Rheb-GTP expression by TSC1, thereby inhibiting the expression of mTOR. Meanwhile, miR-99b targets and downregulates the expression of mTOR, affecting autophagy and facilitating viral replication (145).

Based on the autophagy-dependent viral replication model, targeting rotavirus NSP4 or host calcium-CAMKK2-AMPK signaling represents a promising therapeutic direction. Inhibitors of calcium release or AMPK activation have shown efficacy in MA104 cells and suckling mouse models by reducing autophagic activation and viral replication but require further validation in clinical trials to assess safety in infants and potential impact on intestinal mucosal homeostasis.

### 4.7 Zika virus

The Zika virus exists as virions with a diameter of approximately 50nm. During the process of Zika virus morphogenesis, phosphatidylserine (PS), which is derived from host cells, is carried on the surface of the Zika virus. PS is capable of directly interacting with the lipid receptor TIM (T cell immunoglobulin mucin) located on the cell surface and subsequently entering cells via endocytosis. Moreover, PS can also interact with another cell receptor, namely TAM (receptor tyrosine kinases including TYRO3, AXL, and MER), through the bridging molecules Growth Arrest-Specific 6 (GAS6) or Protein S (PROS), thereby facilitating its entry into host cells (146–149).

ZIKV’s interaction with autophagy conforms to the “host protection-pathogen exploitation” dichotomy framework, involving classical autophagy, selective autophagy, and secretory autophagy. Current studies are primarily based on fetal neural stem cells (FNSCs), trophoblast cells, human dermal fibroblasts, and pregnant mouse models, with moderate consistency across models—autophagy’s role varies by cell type and infection stage, with prominent pro-viral functions in neural and placental cells. On the one hand, the internalized Zika virus is enwrapped by vesicles, thereby forming primary endosomes. The acidic environment present within the lumen of the endosomes will induce conformational alterations in the glycoproteins located on the surface of the Zika virus particles. Such alterations will then promote the fusion of the viral envelope with the endosomal membrane, consequently resulting in the release of nucleic acids (147).

ZIKV’s TIM/TAM-mediated entry and ER remodeling-dependent replication processes intersect with autophagic pathways, where viral NS4A/NS4B proteins and host TLR3-MyD88/TRIF-Beclin 1 axis coregulate autophagic innate immune signals, including type I interferon production. This pathway crosstalk has been validated in FNSCs and trophoblast cells, confirming stage-specific autophagy modulation for viral benefit. The endoplasmic reticulum provides a membrane platform for the replication and translation of the Zika virus. The genome of the Zika virus is a single-stranded positive-sense RNA with only one open reading frame, which is translated into a long polyprotein. This polyprotein can be cleaved by the host Furin protease to form structural protein capsid (C), membrane precursor (PRM), envelope (E), and non-structural proteins (NS1, NS2A, NS2B, NS3, NS4A, NS4B, and NS5) (150). Among them, the non-structural proteins NS4A and NS4B can promote the remodeling of the endoplasmic reticulum membrane, change the curvature of the endoplasmic reticulum, and thus enable the assembly of Zika virus particles (151, 152). Immature virus particles are transported to the Golgi apparatus via vesicles and undergo processing therein to form mature virus particles, which are subsequently secreted out of the cells through exocytosis. Nevertheless, when excessive viral proteins are synthesized, endoplasmic reticulum stress will be induced, and consequently, the unfolded protein response (UPR) pathway will be activated. This activation leads to a reduction in the amount of proteins flowing into the endoplasmic reticulum, thereby inhibiting the assembly of the Zika virus. In the event that the endoplasmic reticulum accumulates an excessive load of viral proteins and becomes overburdened, the UPR will directly trigger selective autophagy to degrade the proteins of the Zika virus (153). Nevertheless, the research carried out by Turpin et al. revealed that ZIKV is capable of influencing the unfolded protein response (UPR) signaling pathway through downregulating the expression of glucose-regulated protein 78 (GRP78). This action maintains the endoplasmic reticulum in a continuous state of stress, enabling ZIKV to evade the host’s defense system and facilitating the replication and assembly of the Zika virus (154). In addition, Zika can also utilize proteases to directly cleave family with sequence similarity 134, member B (FAM134B), specifically inhibiting the occurrence of selective autophagy (155).

The autophagic response induced by ZIKV is predominantly classical, regulated by the PI3K-Akt-mTOR, TLR3-MyD88/TRIF-Beclin 1, and UPR signaling axes. Research controversies exist: FNSC studies report that NS4A/NS4B-induced autophagy promotes viral replication, while nerve cell and glial cell studies demonstrate that ZIKV activates mTORC1/2 to suppress autophagy in the late infection stage. This discrepancy is attributed to the infection stage (early induction vs. late suppression) and cell type-specific differences in autophagic regulatory networks. Additionally, trophoblast cell studies show secretory autophagy facilitates vertical transmission, while dermal fibroblast studies highlight autophagy’s antiviral potential via protein degradation. On the other hand, the Zika virus that enters the cells can be recognized by different pattern recognition receptors. Among them, Toll - like receptor 3 (TLR3) has been reported more frequently (147, 156). When TLR3 is activated by the Zika virus, the TLR3 signaling pathway is initiated, which can induce the body to produce type I interferons. The research by Huang et al. found that the activation of the type I interferon signaling pathway is associated with autophagy (157). Furthermore, the activated Toll-like receptor 3 (TLR3) is capable of recruiting the adaptor proteins, namely Myeloid Differentiation Primary Response Protein (MyD88) and TIR domain-containing adaptor inducing interferon-β protein (TRIF). It has been verified through research that MyD88 and TRIF can undergo co-immunoprecipitation with Beclin 1. The TLR signaling pathway augments the interaction among MYD88, TRIF, and Beclin 1, thereby dissociating Beclin 1 from the B-cell lymphoma 2 (Bcl-2)-Beclin 1 complex (158). Beclin1 is a component of the phosphatidylinositol 3-kinase (PI3K) complex. This complex is phosphorylated to form phosphatidylinositol 3-phosphate (PI3P), which is essential for the formation of autophagosomes in the classic autophagy pathway (159).

Autophagy can not only directly degrade the nucleic acids and proteins of Zika virus but also provide assistance for the replication and vertical transmission of Zika virus, which is of particular significance for Zika virus infection. Up to now, it has been verified that Zika infection can induce autophagy within multiple types of cells, such as fetal neural stem cells (160), human dermal fibroblasts (161), trophoblast cells and umbilical vein endothelial cells (162). Moreover, the replication level of Zika can be significantly enhanced after being treated with autophagy inducers. Furthermore, it has been revealed through research that the occurrence of autophagy subsequent to the entry of Zika into the host is not merely a passive response of the organism but may also be the consequence of the proactive actions exerted by Zika for the purpose of facilitating its own replication. The ankyrin repeat and sterile α motif domain containing 4b (ANKS4B) protein serves as a negative regulator in the process of autophagy. Nevertheless, in both *in vivo* and *in vitro* Zika infection experiments, the expression level of ANKS4B was found to be downregulated. Moreover, the viral replication level was significantly elevated in cells with ANKS4B deficiency. These findings suggest that Zika might augment the autophagic flux by downregulating ANKS4B, thereby facilitating an increase in viral replication (163). Nonetheless, at present, research on the mechanism through which Zika virus attains high - level replication via the autophagy pathway remains limited. Liang conducted an experiment where human fetal neural stem cells (FNSCs) were infected with Zika virus. After screening all the structural and non - structural proteins of the Zika virus, it was discovered that NS4A and NS4B act in a coordinated manner on the PI3K - Akt - mTOR signaling pathway, a crucial regulator of autophagy. Specifically, they inhibit the upstream PI3K signal. Concurrently, they attenuate the phosphorylation of Akt at Thr308 and Ser473, thereby obstructing the activation of Akt. This inhibitory effect on the host’s Akt - mTOR signal transduction leads to the upregulation of autophagy, which in turn promotes an increase in viral replication (157, 160). However, further research by Sahoo et al. found that autophagy was only transiently induced in the early and middle stages of Zika virus infection in nerve cells and glial cells. In the later stage of infection, mTORC1 and mTORC2 were activated by the Zika virus and negatively regulated autophagy, resulting in the accumulation of the virus. Depleting the key components of these two complexes by using siRNA had an impact on the replication and protein expression of the Zika virus, demonstrating that mTORC1 and mTORC2 are essential for the replication and protein expression of the Zika virus (164). Nevertheless, the regulation of autophagic flux following Zika infection cannot be solely ascribed to Zika. The activation of autophagy in the early stage is induced by multiple pathways. Sun et al. identified a specific salivary protein of female mosquitoes, namely Aedes aegypti venom allergen-1 (AAVA-1). AAVA-1 is capable of entering human immune cells in a manner dependent on Ras Homolog Family Member A (RhoA). Subsequently, it undergoes intracellular transport from endosomes to mitochondria and sequesters Leucine Rich Pentatricopeptide Repeat Containing (LRPPRC), which functions as an inhibitor of Beclin 1, onto the mitochondria, thereby facilitating the activation of autophagy (165). This finding suggests that Zika infection is capable of triggering autophagy in multiple types of cells. During the early stage of infection, Zika virus facilitates its own replication by inducing autophagy. Nevertheless, in the subsequent stage, it becomes necessary for the virus to evade the host’s phagocytic process and suppress the level of autophagy. The specific mechanisms underlying these phenomena await further clarification.

The dual role of autophagy in ZIKV infection reflects the complex interplay between host selective autophagy (viral protein degradation) and viral exploitation of autophagy for replication, immune evasion, and vertical transmission. Consistent across key models (FNSCs, trophoblast cells, pregnant mice), autophagy is a critical mediator of ZIKV’s neurotropism and placental traversal. An unresolved controversy is how to balance the inhibition of pro-viral secretory autophagy and preservation of antiviral degradative autophagy in therapeutic strategies, particularly for preventing fetal microcephaly. It is worth noting that autophagy is not only associated with the viral yield of the Zika virus but also plays a role in promoting the vertical transmission of the Zika virus as well as its infection within the placenta. Cao et al. demonstrated that the deletion of the key autophagy gene Atg16L1 in trophoblast cells can restrain the vertical transmission of the Zika virus. Additionally, the application of the autophagy-inhibiting drug hydroxychloroquine in pregnant mice infected with the Zika virus can mitigate the damage to fetuses and placentas (162, 166). In addition, secretory autophagy might also serve as one of the pathways by which Zika traverses the placental barrier. Zika gains entry into endosomes via endocytosis and subsequently has the potential to form autophagosomes. Once the autophagosomes fuse with lysosomes, Zika is subject to degradation. However, Zika may alternatively impede the fusion of autophagosomes with lysosomes and instead give rise to exosomes. As reported, the exosomes released by cells infected with Zika contain Zika proteins, among which NS1 is included. These exosomes might offer a shielding barrier for Zika, allowing it to cross the placental barrier while remaining protected from attacks by the maternal immune system (167). Yuan et al. found that trehalose can promote the fusion of autophagosomes with lysosomes, prevent Zika from entering the exosome pathway, and expose it to the maternal immune system, thus leading to its degradation (168). Currently, the exact mechanism by which Zika crosses the placental barrier remains unclear. However, given that Zika has a neurotropic property, it may damage the fetal brain when it penetrates the placenta. Therefore, it is necessary to balance secretory autophagy and degradative autophagy in the prevention and treatment of microcephaly (169).

Based on the stage- and cell type-specific autophagy model, targeting ZIKV NS4A/NS4B or host mTOR/Beclin 1 signaling represents a promising therapeutic direction. Agents like trehalose, which promote autophagosome-lysosome fusion, have shown efficacy in pregnant mouse models by reducing vertical transmission and fetal brain damage, while inhibitors of secretory autophagy may complement antiviral effects. These strategies require further validation in clinical trials to assess safety in pregnant individuals and long-term neurodevelopmental outcomes in infants.

## 5 Autophagy in highly concerned severe zoonotic infectious diseases

### 5.1 Brucellosis

**Figure 5:**
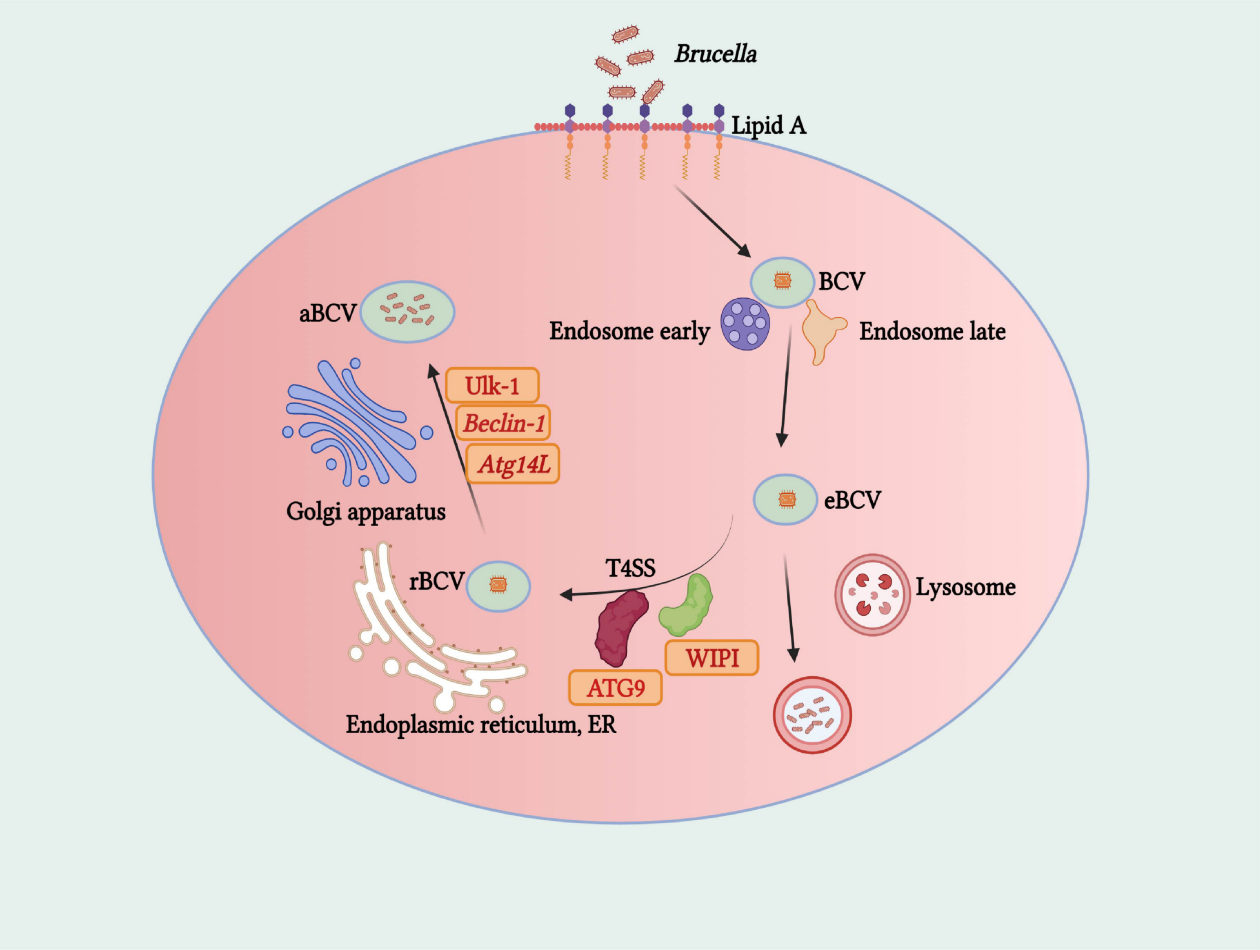
Non - classical autophagy pathway of Brucella. Brucella enters host cells and forms Brucella - containing vacuoles (BCV) through the interaction between lipid rafts and the cell membrane. The BCV interacts with early endosomes (EE) and late endosomes (LE), and partially fuses with lysosomes to form endosomal Brucella-containing vacuoles (eBCV). The eBCV that escapes lysosomal degradation activates the type IV secretion system (T4SS), mediating the interaction between effector proteins and the endoplasmic reticulum exit site (ERES). Then it reaches the endoplasmic reticulum and fuses with it in a Sar1- and Rab2-dependent manner to form repetitive Brucella - containing vacuoles (rBCV). In the late stage of the intracellular cycle of Brucella, the rBCV derived from the endoplasmic reticulum transforms into autophagic Brucella - containing vacuoles (aBCV). Brucella promotes the accumulation of endoplasmic- reticulum-derived membranes on eBCV and converts it into rBCV by disrupting the autophagy - related proteins ATG9 and WIPI. Subsequently, aBCV is formed with the participation of the autophagy-related protein Ulk-1, the autophagy - related genes Beclin-1 and Atg14L, and the activation of PI3-K.

Brucellosis is a zoonotic infectious disease caused by *Brucella*. It is characterized by abortion in pregnant animals and reproductive dysfunction (170). *Brucella* is a Gram - negative facultative intracellular bacterium. It not only has the function of immune evasion but can also inhabit host cells for a long time, leading to chronic and persistent infections in the body (171). This pathogen’s interaction with autophagy aligns with the “host protection-pathogen exploitation” dichotomy framework and involves non-canonical autophagy. Current studies are primarily based on RAW264.7 cells, primary macrophages, and mouse models, with high consistency in key findings across different models. Once *Brucella* invades host cells, the autophagy pathway is activated. Notably, *Brucella* is capable of leveraging its unique evasion mechanisms to modulate the autophagic process. Consequently, it multiplies extensively within host cells and establishes a chronic infection. This phenomenon suggests that the autophagic response plays a facilitative role in the survival and replication of *Brucella* within the cells (172). Nevertheless, research findings indicate that the cellular autophagy pathway induced by *Brucella* pertains to a form of non - classical autophagy, which exhibits certain discrepancies when compared to classical autophagy (173).(**Figure 5**).

*Brucella*’s invasion and intracellular trafficking process intersects with autophagic pathways, and its lipid raft-mediated entry and Type IV secretion system (T4SS) are critical for regulating autophagic innate immune signals, such as the cGAS-STING pathway. This invasion and trafficking cascade has been validated in both cell lines and primary cell models, showing good reproducibility, though *in vivo* studies have further confirmed the physiological relevance of BCV maturation dynamics. *Brucella* enters host cells and forms (BCV) through the interaction between lipid rafts and the cell membrane (174). BCV engages in interactions with early endosomes (EE) and late endosomes (LE), and undergoes partial fusion with lysosomes, thereby giving rise to the formation of endosomal *Brucella* - containing vacuole (eBCV). The eBCV, which evades lysosomal degradation, activates the *Brucella* type IV secretion system (T4SS). This activation enables the mediation of interactions between effector proteins and the endoplasmic reticulum exit site (ERES). Subsequently, the eBCV translocates to the endoplasmic reticulum and fuses with it in a manner that is contingent upon Sar1 and Rab2, ultimately resulting in the generation of repetitive *Brucella* - containing vacuole (rBCV) (175). During the later stage of the intracellular cycle of *Brucella*, the rBCV originating from the endoplasmic reticulum undergoes a transformation into autophagic *Brucella* - containing vacuole (aBCV). This transformation event suggests that the non - classical autophagy induced by *Brucella* shares common upstream regulatory pathways with classical autophagy. Evidently, this non - classical autophagic process plays a crucial role in the intracellular cycle of *Brucella* and significantly promotes its intracellular dissemination.

The non-canonical autophagy induced by *Brucella* is characterized by ATG5/ATG7 independence, with distinct regulatory requirements for different stages of BCV maturation. Research controversies exist: early studies using RAW264.7 cells proposed that eBCV-to-rBCV conversion is completely independent of core autophagy initiators, while recent primary macrophage studies suggest partial involvement of Beclin-1 in late-stage aBCV formation. This discrepancy may arise from the simplified autophagic regulatory network in immortalized cell lines compared to primary cells with intact immune signaling. Findings from studies have demonstrated that the maturation process of eBCV into rBCV does not necessitate the involvement of the autophagy - initiating protein Ulk - 1, the autophagy - related gene Beclin - 1, as well as the autophagy - elongation proteins ATG5, ATG7, ATG16L, and LC3B. *Brucella* promotes the accumulation of endoplasmic reticulum - derived membranes on eBCV and converts it into rBCV by disrupting autophagy - related proteins ATG9 and WIPI. This is functionally distinct from classical autophagy (173). In contrast, the formation of autophagic *Brucella* - containing vacuole (aBCV) necessitates the involvement of the autophagy - associated protein Ulk - 1, along with the autophagy - related genes Beclin - 1 and Atg14L. Additionally, the activation of phosphatidylinositol 3 - kinase (PI3 - K) is also required. Intriguingly, this process shows no association with the autophagy - elongation proteins ATG5, ATG4B, ATG7, ATG16L, and LC3B (176). This suggests that the generation of aBCV is not contingent upon the autophagy - elongation complex. Rather, it is achieved through the interaction between the locally - located Beclin 1 - ATG14L complex within the endoplasmic reticulum and rBCV. The formation of aBCV not only serves as an indicator of the completion of the intracellular cycle of *Brucella* but also acts as a promoter for its subsequent chronic infection. During distinct phases of the intracellular cycle, *Brucella* selectively exploits the autophagy - initiation complex to undermine the host’s pathogen - clearance mechanisms, thereby facilitating its own chronic infection process.

**Figure 6:**
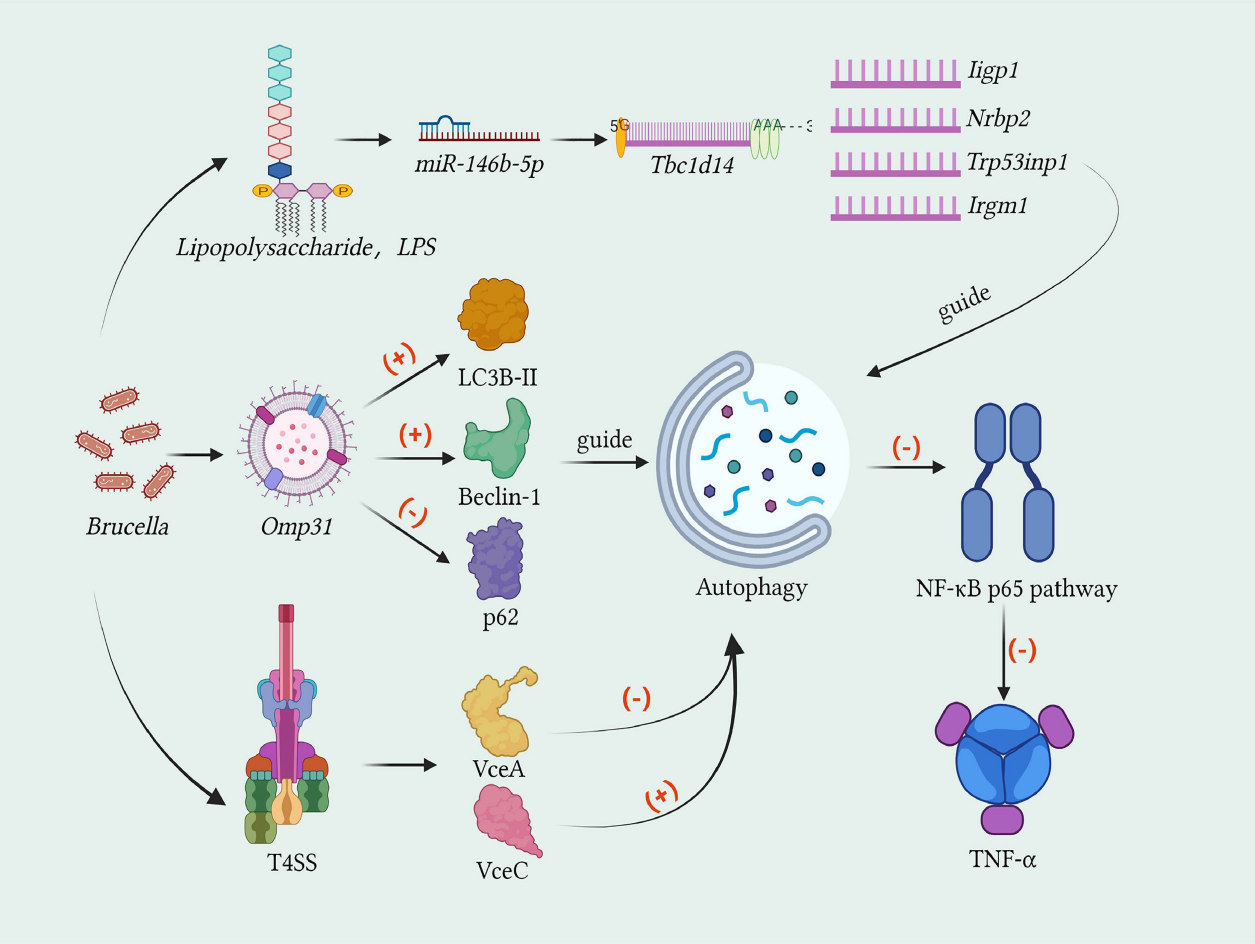
Autophagy activation and its regulatory pathways in brucella melitensis. 1)The lipopolysaccharide (LPS) of Brucella melitensis (B. melitensis) affects the induced cellular autophagy response by regulating miR-146b-5p and its target Tbc1d14, as well as autophagy-related genes Iigp1, Nrbp2, Trp53inp1, and Irgm1. 2)B. melitensis Omp31 can induce the occurrence of cellular autophagy by promoting the expression of LC3B-II and Beclin-1 proteins and inhibiting the level of p62 protein. The autophagy induced by it inhibits the expression of TNF-α by negatively regulating the NF-κB p65 signaling pathway. 3)The Type IV secretory system (T4SS) encoded by the VirB promoter serves as an important virulence factor of Brucella. Its effector factors VceA and VceC play a promoting and an inhibitory role, respectively, in cellular autophagy.

The pro-bacterial role of non-canonical autophagy in *Brucella* infection reflects the evolutionary interplay between host xenophagic clearance and bacterial autophagy hijacking. Consistent across cell lines, primary cells, and mouse models, this non-canonical autophagy provides a protective niche for *Brucella* survival and replication. An unresolved controversy is whether host xenophagy can be reactivated to counteract *Brucella* during chronic infection, as current evidence primarily supports bacterial exploitation of autophagy. Research has found that the lipopolysaccharide (LPS) of *Brucella melitensis* (*B. melitensis*) can influence the cellular autophagy response induced by it through regulating miR - 146b - 5p and its target Tbc1d14, as well as autophagy - related genes including Iigp1, Nrbp2, Trp53inp1 and Irgm1 (177). (**Figure 6**) *B. melitensis* Omp31 has been demonstrated to induce cellular autophagy. It exerts this effect by upregulating the expression of LC3B-II and Beclin-1 proteins and downregulating the level of p62 protein. Mechanistically, the autophagy induced by *B. melitensis* Omp31 negatively regulates the NF-κB p65 signaling pathway, thereby suppressing the expression of TNF-α (178). The Type IV secretory system (T4SS) encoded by the VirB promoter is a pivotal virulence factor in *Brucella*. The effector molecules VceA and VceC within this system have been shown to play essential and multifaceted roles in modulating cellular autophagy and apoptosis. These functions not only influence the intracellular survival and replication of *Brucella* but also have profound implications for the host - pathogen interaction dynamics, thereby contributing to the overall pathogenicity of *Brucella* infections (179). Deng X and colleagues discovered that the deletion of the VirB promoter of the *Brucella* type IV secretion system (T4SS) leads to a reduction in bacterial virulence and an enhancement of autophagy (180). *Brucella* triggers the phosphorylation of p38 MAP kinase and induces cellular autophagy in a T4SS - dependent fashion. This phenomenon suggests that the cross - point between the MAP kinase pathway and the autophagy mechanism represents a pivotal element in the intracellular life cycle of *Brucella*. It implies that the crosstalk between these two biological processes likely plays a significant role in regulating various aspects of *Brucella’s* survival, replication, and persistence within host cells, which is of great importance for understanding the pathogenic mechanism of *Brucella* and developing potential intervention strategies (181).

Based on the non-canonical autophagy-centered interaction model, targeting bacterial T4SS or host PI3-K/Beclin-1 signaling represents a promising therapeutic direction for brucellosis. Strategies inhibiting non-canonical autophagy have been validated in RAW264.7 cells and mouse models, showing reduced *Brucella* intracellular load, but require further confirmation in primary human cells and clinical settings to assess safety and efficacy (182). A deeper understanding of the underlying molecular mechanisms by which *Brucella* induces autophagy in host cells will further facilitate in - depth exploration of the intracellular parasitism and immune evasion mechanisms of *Brucella*. This provides a profound theoretical basis and direction for the prevention and treatment of clinical brucellosis.

### 5.2 Anthrax

The causative agent of anthrax is Bacillus anthracis. This bacterium generates two crucial virulence factors: lethal toxin (LT) and edema toxin. Lethal toxin consists of lethal factor (LF) and protective antigen (PA), which is responsible for cytoplasmic transport, while edema toxin is composed of edema factor (EF) and PA. PA has the ability to bind and interact with two anthrax toxin receptors, namely anthrax toxin receptor 1 (ANTXR1) and anthrax toxin receptor 2 (ANTXR2) (183). These toxins are the main causes of the clinical manifestations of anthrax. Once they enter the cytoplasm, they are cytotoxic to host cells, which can induce circulatory shock, such as cardiovascular complications, and even lead to death.

B. anthracis-induced autophagy adheres to the “host protection-pathogen exploitation” dichotomy framework, primarily involving classical autophagy and Cathepsin B (CTSB)-dependent autophagic flux. Current studies are predominantly based on mammalian cell lines (e.g., HeLa, cardiomyocytes), human polymorphonuclear leukocytes, and mouse models, with high consistency in core findings—autophagy functions as both a host defense mechanism against bacterial clearance and a facilitator of toxin-mediated pathogenicity. LT is the main virulence factor of anthrax. Studies have shown that exposure to LT can lead to increased ROS accumulation, myocardial contractile dysfunction, impaired intracellular Ca2+ handling, decreased mitochondrial membrane potential, enhanced ubiquitination, and significant autophagy (184).

The interaction between B. anthracis toxins (LT, PA) and host ANTXR1/ANTXR2 receptors intersects with autophagic pathways, where PA-initiated autophagy and CTSB-dependent autophagic flux coregulate toxin delivery and host immune responses. This pathway crosstalk has been validated in multiple mammalian cell models, confirming the dual role of autophagy in anthrax pathogenesis. Some studies have shown that LT and PA can induce autophagy in mammalian cells and may be related to the cellular defense mechanisms against LT - mediated toxemia. Furthermore, Xia Shunde et al. have demonstrated that the binding of Protective Antigen (PA) to either anthrax toxin receptor 1 (ANTXR1) or anthrax toxin receptor 2 (ANTXR2) can initiate autophagy, which consequently facilitates the cytoplasmic delivery of LF associated with ANTXR2. Moreover, they have identified that the cytoplasmic delivery of LF by ANTXR2 is mediated by Cathepsin B (CTSB). Notably, in cells with a deficiency in CTSB, the autophagic flux is significantly delayed, suggesting that the CTSB - dependent autophagic flux plays a crucial role in enhancing the cytoplasmic delivery of LF mediated by ANTXR2. This finding highlights the intricate interplay among these molecular components and their significant implications for understanding the pathogenic mechanisms underlying anthrax and related cellular responses (185).

The autophagic response induced by B. anthracis toxins is classical, regulated by the ANTXR-PA-CTSB and TLR4-dependent signaling axes. Research controversies are minimal, though minor discrepancies exist regarding autophagy’s net effect in cardiac cells: some studies suggest autophagy exacerbates LT-induced myocardial damage, while others imply that targeted autophagy inhibition alleviates pathological effects. This subtle difference is attributed to the dosage of LT exposure and the activation stage of autophagic flux (early induction vs. late impairment). In the context of the abnormal myocardial cell contraction and intracellular Ca2+ dysregulation in patients caused by lethal toxin (LT), some studies have indicated that inhibiting autophagy might alleviate the pathological effects of LT on the heart. For instance, the knockout of Toll-like receptor 4 (TLR4) might prevent LT-induced myocardial contraction and intracellular calcium ion abnormalities through autophagy-related mechanisms (186). Additionally, cellular catalase might also play a role in preventing LT-induced myocardial contraction and intracellular calcium ion abnormalities by regulating autophagy and mitochondrial function (187).

The dual role of autophagy in B. anthracis infection reflects the balance between host autophagy-mediated bacterial clearance (e.g., in neutrophils) and bacterial exploitation of autophagy for toxin delivery (e.g., in ANTXR2-expressing cells). Consistent across cell and animal models, autophagy is a critical mediator of both host defense and toxin-induced pathogenicity. An unresolved question is how to selectively target the pro-pathogenic autophagic pathways (e.g., CTSB-dependent flux) while preserving the host’s antiviral autophagic defense. There are also studies that have demonstrated, by treating human polymorphonuclear leukocytes with 3-methyladenine (an autophagy inhibitor), that autophagy in human neutrophils is an important mechanism for killing Bacillus anthracis (188). All the above studies have shown that the research on autophagy is of great significance for studying the pathogenesis, treatment, and management methods of anthrax. The autophagy mechanism triggered by Bacillus anthracis infection remains unclear. Although the pathogenicity of Bacillus anthracis can be influenced by some autophagy regulators, there is still a vast space for research in terms of the specific autophagy pathways, the action mechanisms, and the reliable treatment methods for anthrax.

Based on the CTSB-dependent autophagic flux model, targeting B. anthracis PA or host CTSB/ANTXR2 signaling represents a promising therapeutic direction. Autophagy inhibitors (e.g., 3-methyladenine) have shown efficacy in human polymorphonuclear leukocytes and mouse models by enhancing bacterial clearance and alleviating LT-induced myocardial damage, but require further validation in clinical settings to assess safety and potential impact on overall host immunity.

### 5.3 Avian influenza

Influenza pandemics are caused by avian influenza viruses, which pose pandemic and zoonotic threats to humans. There are multiple subtypes of avian influenza viruses. Among them, H5N1 and H9N2 are candidate viruses for pandemics. Human H5N1 disease has a mortality rate exceeding 60%, threatening human public health security. H9N2 has been highly prevalent among poultry in many countries and there have been repeated cases of human infections (189).

Avian influenza virus (AIV)-induced autophagy adheres to the “host protection-pathogen exploitation” dichotomy framework, with subtype-specific modulation of classical autophagy and autophagic flux. Current studies are primarily based on alveolar epithelial cells, A549 cells, and mouse models, with high consistency in core findings—autophagy’s role varies by AIV subtype, exerting pro-pathogenic effects in H5N1 and H9N2/G1 infections while serving as an antiviral mechanism in host defense against certain subtypes. Autophagy plays varying roles in different subtypes of avian influenza viruses. When compared with the seasonal influenza A subtype H1N1, the H9N2/G1 subtype induces autophagy to a greater degree, which is concomitantly accompanied by the excessive induction of cytokines. Moreover, both the H5N1 and H9N2/G1 subtypes can trigger significantly higher levels of cytokines and chemokines, including tumor necrosis factor-α, type I interferon, chemokine ligand 2 (CCL2), CCL3, CCL5, and chemokine Ligand-10 (CXCL10) (190, 191). It is likely that these phenomena are associated with the pathogenic consequences of avian influenza virus infections (192).

The interaction between AIV (H5N1, H9N2/G1) and host cells intersects with autophagic pathways, where subtype-specific autophagy induction (H9N2/G1 > H1N1) and autophagic flux modulation coregulate cytokine production (e.g., CXCL10, IFN-α) and innate immune responses. This pathway crosstalk has been validated in A549 cells and alveolar epithelial cells, confirming the link between autophagy and AIV-induced immunopathology. Since autophagy plays a role in inducing cell death (193), it may also lead to cytokine imbalance. Studies by Luo Qingyi et al. have shown that H9N2/G1 is a stronger autophagy inducer compared to H1N1. Moreover, after viral infection, autophagy plays a role in the production of CXCL10 and IFN-α. They have demonstrated that after inhibiting the autophagy pathway by knocking down Atg5, the protein levels of CXCL10 and IFN-α induced by H9N2/G1 were reduced by 50%. However, the detailed mechanism by which H9N2/G1 induces CXCL10 and IFN-α through autophagy requires further investigation (194).

The autophagic response induced by AIV is subtype-specific and classical, regulated by the mTOR, NF-κB, P38-MAPK, JNK, and PI3-K signaling axes. Research controversies exist: some studies report that autophagy promotes AIV replication via JNK activation, while others demonstrate that host autophagy limits viral replication. This discrepancy is attributed to AIV subtype differences (H5N1 vs. H9N2 vs. H1N1) and the stage of infection (early antiviral vs. late pro-pathogenic). H5N1 is one of the few avian influenza viruses that can cross the species barrier to infect humans. The main target of the H5N1 virus is alveolar epithelial cells (195). Since the mechanism underlying lung injury caused by H5N1 infection remains unclear, acute respiratory distress syndrome (ARDS) is the main cause of death among the affected individuals (196). Ma Jianhui et al. have demonstrated that H5N1 can lead to autophagy-mediated cell death by inhibiting the mTOR signaling pathway (197). Subsequently, Pan Hong et al. indicated that autophagy mediates the lung inflammation induced by H5N1 pseudotyped particles through the NF-κB and P38-MAPK signaling pathways, which might point out the direction for clarifying the mechanism underlying lung injury caused by H5N1 infection (198); Later, Sun Yang et al. showed that inhibiting cellular autophagy could improve the acute lung injury resulting from H5N1 virus infection (199). Distinct influenza A avian influenza subtypes exert diverse effects on autophagy. Specifically, the H5N1 virus has been found to induce functional autophagy, whereas the H9N2 and H1N1 viruses are capable of blocking the autophagic flux within cells. These disparities can be attributed to the alternate activation of the c-Jun terminal kinase (JNK) and phosphatidylinositol 3-kinase (PI3-K) pathways. Notably, it has been verified that the activation of the JNK pathway can enhance the replication of influenza A avian influenza viruses (200).

The dual role of autophagy in AIV infection reflects the subtype-specific interplay between host autophagy-mediated viral restriction and viral exploitation of autophagy for immunopathology and replication. Consistent across cell and animal models, autophagy is a key mediator of AIV-induced lung injury and cytokine storm. An unresolved controversy is how to develop subtype-specific autophagy-targeted therapies, as the net effect of autophagy varies significantly between high-pathogenic (H5N1) and low-pathogenic (H9N2) AIV subtypes. The latest research shows that mammalian cells can utilize the autophagy process to limit the replication of avian influenza viruses (201). At present, the research on the interaction between avian influenza viruses and autophagy is still incomplete. Studying autophagy in avian influenza is of great significance for the pathogenesis of avian influenza, the replication mechanism of its viruses, and the treatment of the disease.

Based on the subtype-specific autophagy modulation model, targeting AIV-induced JNK/PI3-K signaling or host mTOR/NF-κB pathways represents a promising therapeutic direction. Autophagy inhibitors have shown efficacy in mouse models of H5N1-induced acute lung injury by reducing inflammation and improving survival, while strategies enhancing autophagy may be beneficial for limiting H9N2 replication. These approaches require further validation in preclinical studies to assess subtype-specific efficacy and safety.

### 5.4 Rabies

Rabies is a fatal viral disease caused by rabies virus infection. The rabies virus (Rabies virus, RABV) mainly invades the central nervous system and can induce different degrees of cellular autophagy through different pathways. These differences are related to the virus strains, infected cells and RABV proteins.

RABV-induced autophagy conforms to the “host protection-pathogen exploitation” dichotomy framework, featuring strain-specific and cell type-dependent modulation of classical autophagy, incomplete autophagy, and mitophagy. Current studies are primarily based on neuroblastoma cell lines (SK, NA), microglial cells (BV2), and mouse models, with high consistency in core findings—autophagy induction varies by RABV strain pathogenicity and host cell type, exerting dual effects on viral replication and neuroprotection. Differences in virus strains lead to variations in the degree of autophagy. The attenuated Chinese rabies vaccine strain (SRV9) results in the accumulation of more autophagosomes than the pathogenic challenge virus standard 11 (CVS-11) (202, 203).

The interaction between RABV and host neural cells intersects with diverse autophagic pathways, where viral proteins (P5, G, rL-RVG) and host signaling axes (AMPK-MTOR, PERK/eIF2α/CHOP) coregulate autophagic flux and innate immune responses (e.g., type I interferon production). This pathway crosstalk has been validated in multiple neural and non-neural cell models, confirming the specificity of RABV protein-mediated autophagy induction. The degree of autophagy varies depending on the infected cells. RABV induces incomplete autophagy in mouse microglial cells (BV2), and the intensity of autophagy is positively correlated with the viral load (204). Infection with wild - type RABV GD - SH - 01 induces incomplete autophagy in NA cells; while it induces complete autophagy in the human neuroblastoma cell line (SK). It was also found that the AMPK - MTOR pathway is activated in SK cells, and this activation process may be directly related to the M protein of the rabies virus (205).

The autophagic response induced by RABV is heterogeneous (complete vs. incomplete) and classical, regulated by virus-specific pathways including BECN1-CASP2-AMPK, PERK/eIF2α/CHOP, and IRE1/JNK/beclin-1. Research controversies exist: some studies suggest autophagy exerts neuroprotective effects against RABV-induced neural damage, while others report that autophagy enhances viral replication and exacerbates infection. This discrepancy is attributed to RABV strain pathogenicity (attenuated vs. wild-type) and the type of infected cells (neuroblastoma vs. microglia). RABV proteins are closely related to autophagy induction. The RABV phosphoprotein P5 binds to BECN1 and can induce incomplete autophagy through the BECN1 - CASP2 - AMPK - MAPK and BECN1 - CASP2 - AMPK - AKT - MTOR pathways (206, 207). The G protein of RABV plays an important role in the innate and adaptive immunity of the virus. The overexpression of the RABV G protein helps to reduce the immunogenicity of attenuated RABV. For example, in NA cells infected with Hep - dG (attenuated rabies virus strain), the production of type I interferon (IFN) was detected. The autophagy - related proteins SQSTM1 and Irgm1 were significantly upregulated. Among them, Irgm1 can regulate the autophagic flux, act on mitochondria, and thus induce autophagy, that is, it causes mitochondrial dysfunction in cells, and then leads to mitophagy and cellular autophagy (208–210). The glycoprotein rL-RVG of RABV can induce autophagy in human gastric cancer cells SGC-7901 and HGC by activating the PERK/eIF2α/CHOP and IRE1/JNK/beclin-1 signaling pathways through endoplasmic reticulum stress (211, 212).

**Figure 7:**
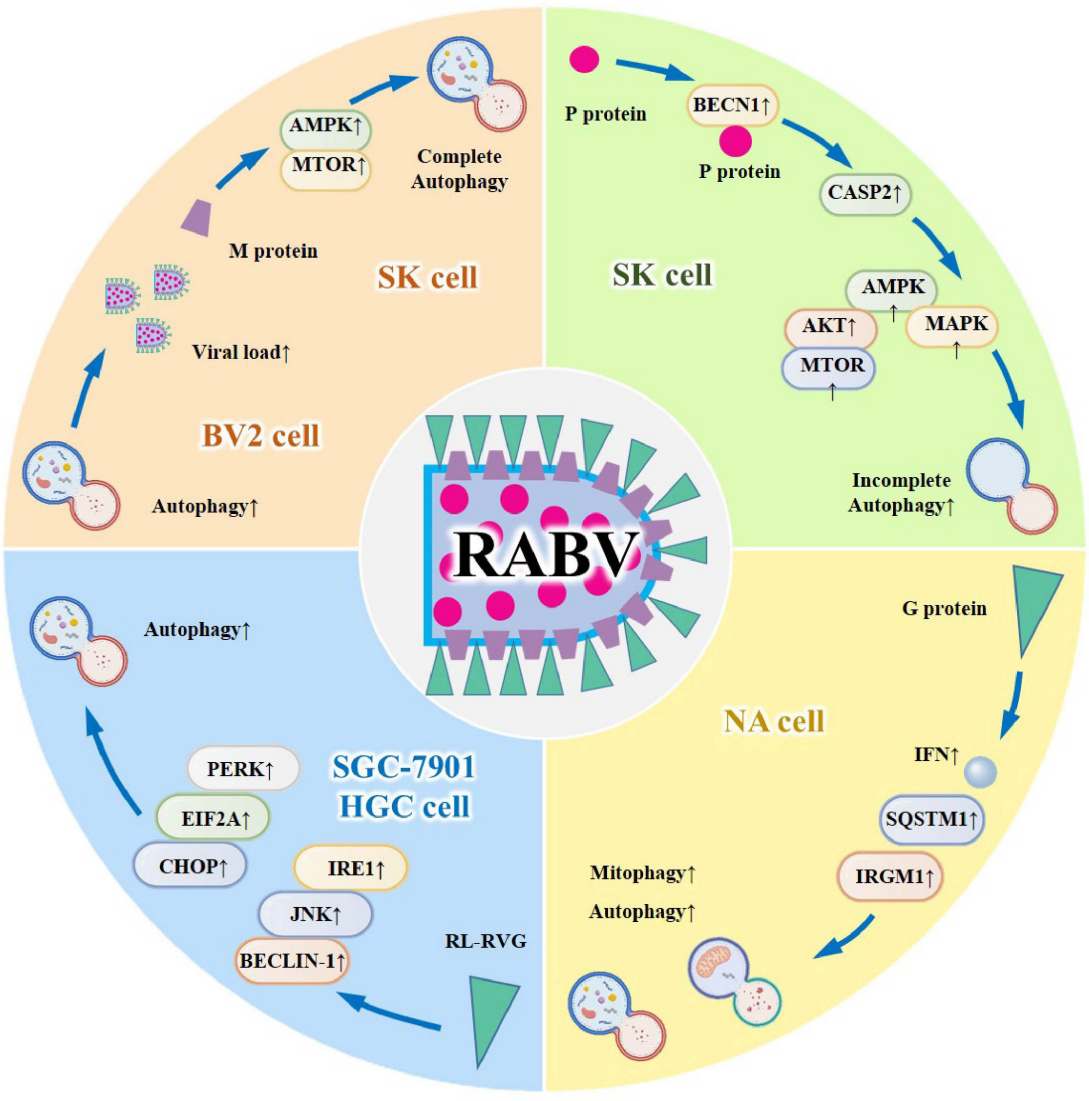
Cell-type-specific interaction between RABV and autophagy and therapeutic implications. This diagram demonstrates that Rabies virus (RABV) interacts with autophagy in a cell - type - specific manner across various cells, including SK, BV2, NA, SGC - 7901, and HGC cells. RABV is capable of inducing either complete or incomplete autophagy through multiple pathways associated with proteins such as P, G, and M, as well as via mechanisms involving molecules like AMPK, MTOR, and PERK. Autophagy exerts an influence on the replication and pathogenicity of RABV, and substances like PGG may inhibit RABV by modulating autophagic processes, thus possessing potential as anti - RABV agents.

The dual role of autophagy in RABV infection reflects the complex interplay between host autophagy-mediated neuroprotection and viral exploitation of autophagy for replication and immune evasion. Consistent across cell and animal models, RABV’s ability to modulate autophagy in a strain- and cell type-specific manner is a key pathogenic feature. An unresolved question is how to leverage autophagy modulation to balance antiviral effects and neuroprotection, given the heterogeneous role of autophagy in different RABV infection contexts. Autophagy also participates in rabies virus infection and affects its replication and/or pathogenicity. Previous studies have demonstrated autophagy in RABV infection models. These studies hypothesized that autophagy may have a neuroprotective effect or lead to increased virus replication and exacerbated infection (205, 207, 210). Currently, there is no clear anti-rabies virus drug. Existing studies have shown that the compound 1,2,3,4,6-penta-O-galloyl-β-d-glucose (PGG) can significantly inhibit the adsorption and entry of early RABV, as well as the gene replication and protein synthesis of RABV through autophagy - dependence. It may be a potential candidate for anti - RABV drugs (213). (**Figure 7**).

Based on the strain- and cell type-specific autophagy model, targeting RABV P5/G proteins or host BECN1/AMPK signaling represents a promising therapeutic direction. Autophagy-dependent inhibitors like PGG have shown efficacy in cell models by suppressing RABV replication, but require further validation in animal models to assess neuroprotective effects and safety. Additionally, strategies targeting Irgm1-mediated mitophagy may offer new avenues for mitigating RABV-induced neural damage.

## 6 Comparative summary table of autophagy’s roles in major zoonotic diseases

To intuitively present the functional differences, core mechanisms, and application potential of autophagy in various zoonotic diseases, the following table systematically summarizes the key research findings of 12 representative diseases, offering references for subsequent mechanism exploration and therapeutic development (**Table 3**).

**Table 3:** Comparative summary table of autophagy's roles in major zoonotic diseases.

Disease name	Type of autophagic role	Core mechanisms	Experimental models	Therapeutic implications
COVID-19	Dual role	Early stage: Xenophagy clears viral particles; Middle-late stage: Viral ORF3a/ORF10 blocks autophagosome maturation and hijacks autophagic membranes for replication	Vero/A549 cells, K18-hACE2 mice, clinical samples	Autophagy inhibitors (GNS561) and mTOR modulators (rapamycin) inhibit viral replication; Vitamin D regulates autophagic balance
SARS	Dual role	Viral nsp6 induces autophagy, and PLpro stabilizes Beclin-1 via deubiquitination; Meanwhile, it blocks autophagosome-lysosome fusion	Vero cells, rhesus macaque model	Inhibitors targeting PLpro can block viral hijacking of autophagy
MERS	Pro-viral role	NSP6 interferes with the SNARE complex to block autophagosome fusion; PLpro interacts with Beclin-1 to promote autophagy initiation	Vero cells, dromedary camel model	SKP2 inhibitors enhance autophagy and inhibit viral replication
Ebola Hemorrhagic Fever	Dual role	Host induces autophagy via ER stress to clear viruses; Viruses utilize autophagy-related proteins to promote intracellular entry	Vero cells, cynomolgus monkey model	AR-12 exerts antiviral effects by inducing autophagy; PDIA3 inhibits viral GP expression via the autophagy-lysosome pathway
Dengue Fever	Pro-viral role	NS4A/NS1 induce ER stress and activate the AMPK/mTOR pathway to promote autophagy; TIM-1 mediates autophagy and viral invasion	Huh7 cells, mouse model	Inhibitors targeting the AMPK pathway reduce viral load
AIDS	Dual role	Early stage: Autophagy is induced to clear HIV; HIV Tat/Nef block autophagosome maturation and inhibit autophagy in macrophages	CD4+ T cells, macrophages, SCID mouse model	Autophagy inducers (rapamycin) enhance HIV clearance; Drugs targeting Nef restore autophagic function
Rotavirus Disease	Pro-viral role	NSP4 activates the CAMKK2-AMPK pathway via calcium signaling to induce autophagy, providing membrane structures for viral replication	MA104 cells, suckling mouse mode	Inhibition of calcium signaling or the AMPK pathway reduces viral replication
Zika Virus	Dual role	Early stage: Autophagy is induced to promote replication; Late stage: mTORC1/2 is activated to inhibit autophagy, avoiding viral clearance	Neural stem cells, placental trophoblasts, pregnant mouse model	Trehalose promotes autophagosome-lysosome fusion to reduce vertical viral transmission; ANKS4B overexpression inhibits autophagy-dependent replication
Brucellosis	Pro-bacterial role	Induces non-canonical autophagy (ATG5/ATG7-independent) and regulates autophagic pathways via T4SS effectors VceA/VceC	RAW264.7 cells, mouse model	Inhibitors targeting T4SS or non-canonical autophagic pathways suppress intracellular bacterial survival
Anthrax	Dual role	Anthrax toxin induces autophagy involved in host defense; Meanwhile, autophagy promotes intracellular delivery of LF to enhance toxin toxicity	Macrophages, mouse model	CTSB inhibitors block autophagy-mediated LF delivery; Autophagy inhibitors alleviate myocardial injury
Avian Influenza	Subtype-specific dual role	H5N1 induces functional autophagy to promote cell death; H9N2/H1N1 blocks autophagic flux to promote viral replication	MDCK cells, chicken embryos, mouse model	Inhibition of the JNK pathway reduces excessive autophagy induced by H5N1; Targeting the PI3K pathway inhibits H9N2 replication
Rabies	Dual role	Attenuated strains induce complete autophagy to clear viruses; Wild-type strains induce incomplete autophagy to promote viral replication in neural cells	SK-N-SH cells, mouse model	PGG inhibits viral adsorption and replication via an autophagy-dependent pathway; Targeting the PERK pathway regulates autophagy in neural cells
